# Global outlook of the multiplier effect of research and development on environmental sustainability

**DOI:** 10.1371/journal.pone.0291370

**Published:** 2023-09-21

**Authors:** Kow Ansah-Mensah, Adams Osman, Clarke Ebow Yalley, Kofi Adu-Boahen

**Affiliations:** 1 Department of Geography Education, University of Education, Winneba, Ghana; 2 Department of Social Studies, University of Education, Winneba, Ghana; Palestine Economic Policy Research Institute, STATE OF PALESTINE

## Abstract

In a time of severe environmental problems and growing public and private expenditure to promote a sustainable society, studies on the impact of Research and Development (R&D) on environmental sustainability hardly ever exist. This study looked at how R&D promotes the attainment of environmentally sustainable goals, globally. Data at the country level was compiled from the United Nations-Sustainable Development Goals (UN-SDG) database and the World Bank, then processed and analysed using spatial techniques such as spatial merge, autofill, autocorrelation, and geographic weighted regression. Expenditure on R&D and publications in scientific journals had a positive impact on SDGs 8 and 11 for all nations. R&D expenditure on SDG 8 was higher for Southern African countries. The impact of scientific and technical journal articles was greater for Middle East countries. Also, scientific and technical journal articles had greater effects on attainment of SDG 11 in Africa. Exports of high technology from South America and Europe were important for achieving SDG 15. However, the effect of patent and trademark applications had minimal effect on achieving environmentally sustainable goals. The study recommends boosting R&D expenditure by governments and international organisations, particularly in Africa because the multiplier impact extends beyond economic development to environmental sustainability which is necessary for the continent to abate the challenges of climate change.

## Introduction

Attaining the goals of sustainable development (SDGs) is dependent on understanding current global social, economic, cultural, and environmental problems, and devising innovative solutions and policies. Arguably, the basis of knowledge and innovative solutions emanates from science through research and development (R&D) [1]. R&D seeks to acquire findings that are novel, creative, systematic, transferable and reproducible. The contribution of R&D to the development of the world is enormous and diverse spanning improvement in water provision [1] food production, disease and vaccine development [2], clothes/ textiles, industrial production/manufacturing, information technology, space exploration, nature protection/conservation [3], business/commerce and artificial intelligence. An unchecked R&D and innovations have left daunting scares on the earth such as wars, famine, privacy invasions, climate change etc. [4].

Despite the possible drawbacks, the world needs more R&D to enrich and compound its sustainable growth. Significant R&D contributions and outcomes to development are proportional to R&D expenditure [5, 6]. Globally, R&D expenditures to GDP have increased from 2010 (1.6%) to 2020 (1.93%) with wide disparities between the various regions of the world [7]. Studies by [6, 8] indicate that countries which spend more of their GDP on R&D have higher returns in economic growth. On the contrary, not all countries have similar returns of R&D expenditure to GPD, as [6] shows that for every 1% increase in R&D expenditure in UK, France, and Netherlands there is a proportional 1% increase in GDP but in Turkey, Portugal, Iceland, and Austria the same expenditure generates less than 0.5% effects on GDP. The non-uniform outcome of R&D to economic growth presents an opportunity to understand the disparity between countries and help in advocating measures for strategic allocation of R&D expenditure on country basis.

More importantly, R&D studies must address the overemphasis on economic growth at the expense of social and environmental development. A sustainable world transcends economic growth hence the need to understand the effect of R&D on the attainment of non-economic dimensions of sustainable development goals (SDGs). The progress of R&D in ensuring social and environment development needs to be propagated as these dimensions have less monetary outcomes to attract the needed government and private research expenditure. Diving into the gap of limited research on the effects of R&D on non-economic SDGs offers a greater opportunity to also understand the backward effect of SDGs in stimulating R&D.

However, in this study attention was given to the relationship between R&D and environmentally sustainable development goals (ESDGs). The reason is the broad scope of studying social and environmental development concurrently. In addition, rates of environmental degradation are at high-risk levels with ramifications of climate change, disasters and species extinctions on social and economic growth. Thus, it is important to direct more attention to how R&D can help the world in achieving the ESDGs. The current study proposed the following hypothesis;

*H1*: Expenditure on R&D does not affect R&D outputs (Scientific and technical articles, total patent application, total trademark application and high-technology exports)

*H2*: R&D has no significant effect on the attainment of ESDGs

The significance of this study is to increase the advocacy for government and private sector funding for R&D. In percentage terms funds towards R&D globally are low with numbers far lower in the global south [7] but R&D have direct and indirect multiplier effects on the development of countries as well as unaccountable effects on other sectors of a country [9, 10]. The study’s essence is to advocate for an increase in R&D for ESDGs and identify which goals need more funding to accelerate their achievement. It will help in apportioning research funds to SDGs on a country basis as developing countries who need research funds get the least from international donors and their governments. Lastly, to commend the contribution of the scientific community in achieving ESDGs.

### Contextual issues

#### The multiplier effects

According to [11], a multiplier is the ratio of a change in outcome due to a change in government expenditure. Thus, by implication, the resultant effect of a $1 extra expenditure by the government leads to a consequent increase in the overall productivity of the economy. The multiplier concept posits that there is a consequent change in the national income when there is an increase in the exogenous factors. This is because any extra expenditure by the government increases the purchasing power of the population while increasing the productivity of the economy. However, the multiplier effect works in reverse order to; when there is a cut in spending, jobs are lost, and individual expenditure reduces which tends to reduce demand in some parts of the economy [12]. This concept of multiplier has been applied in many related studies such as entrepreneurship, finance, tourism and economy [13, 14]. The multiplier effect is applicable in this study because increased R&D spending will encourage research efforts to produce new knowledge and technology to drive development [15]. In addition, an increase in R&D spending in a country and a sector of the economy has a trickling effect on other sectors and countries.

Moretti et al., [16] found that a 10% increase in R&D expenditure of the government directly affected private firms (5–6%) and increased productivity within the country while indirectly affecting/changing the output of related industries abroad. There is an indirect component of the multiplier that affects other parts of the economy or international community through knowledge sharing from R&D outputs. Concerning the expenditure on R&D, an increase in the budgetary allocation could imply an increase in research activities aimed at improving well-being through innovation and novelty. Thus, the expenditure by the government is accompanied by output through scientific journals which when made available can affect societies and economies towards the achievement of the environment-sustainable development.

#### R&D and development

It is believed that the work of [17] provided the basis for analysing economic growth and knowledge production. Knowledge can be envisaged from the supply side with the production of information/data often transferred at a cost lower than the production cost [17]. While on the demand side knowledge is non-rivalry in consumption as a person’s usage of knowledge does not reduce availability to others [18]. In economic growth and development analysis of countries, innovation through R&D plays a very significant role [19] as investing in R&D is tantamount to investing in a country’s economic growth [20]. Several researchers [21–24] have reported an association between R&D and the development of countries. The type of association and level of effect between R&D and development varies per country [25].

Canada, Finland, France, Italy, Portugal, Turkey, and the USA have a direct linkage between their R&D expenditure and development [26]. A significant positive relationship between R&D and economic growth in OECD countries has been reported [27]. According to [5], the rate of growth in countries like the UK, France and the Netherlands was the same as the rate of R&D expenditure whereas other countries such as Turkey had a lower (0.625%) growth effect. Meanwhile, in countries like Portugal, Iceland and Austria the growth effects were 0.3% and 0.4% for every 1% rise in R&D expenditure. It is not far-fetched to suggest that the multiplier effect of R&D on the development and growth of economies is significantly higher in the global north countries than in the global south countries. That is to indicate that R&D does not affect all economies equally; whiles there could be an entirely negative or backward effect [28]. It is important to know how the multiplier effect influences the attainment of ESDGs.

#### Expenditure on R&D

Research and development (R&D) expenditure plays a significant role in determining the level of innovation of a country in recent times. R&D is any activity to create new knowledge or improve existing ones [29]. There has been a steady increase in R&D expenditure globally with amounts standing at $2.2 trillion as at 2019 [30]. According to the [30], the top 10 and 20 developed countries spent averagely $1.863 trillion and $2.078 trillion in 2019, respectively. The global percentage of GDP to R&D per sub-region was low for Central Asia (0.2%), sub-Saharan Africa (0.4%), South and West Asia (0.6%), Arab States (0.6%), Latin America and the Caribbean (0.7%), Central and Eastern Europe (1.0%), East Asia and the Pacific (2.1%) but high for North America and Western Europe (2.5%) [31].

The situation is not so encouraging on the African continent as investment in R&D by governments is very minimal [32] accounting for only 1.1% (US$22.3 billion) of GDP [33]. Egypt, Kenya, Malawi, Morocco, South Africa and Tunisia spent more than 0.6% of their GDP on R&D [33, 34]. There is significant inequality in R&D expenditure between the global north and south which translates into their economic growth and development. However, world leaders are more willing to spend on R&D during emergencies or pandemics. Following the novel coronavirus, the 2021 R&D expenses in only pharmaceuticals totalled some $238 billion [35]. The government does not need pandemics to spend on R&D but must make a conscious effort to invest in sustainable development studies.

## Materials and methods

### Data sources and processing

The scope of this research was all countries in the world. Data was secured from the United Nations Sustainable Development Goals database. The study downloaded the 2019 datasets as that was the closes year when the research begun. In addition, six SDGs which cover environmentally sustainable goals directly and indirectly [36, 37] were used; decent work and economic growth (SDG 8), sustainable cities and communities (SDG 11), responsible consumption and production (SDG 12), climate action (SDG 13), life below water (SDG 14) and life on land (SDG 15). Targets in SDGs 8, 11, and 12 can address social, economic, and environmental issues [37, 38]. Specifically, SDG 8 have indicators 8.4 on resource efficiency and decoupling economic growth from environmental deterioration, 8.8 on labour and a safe workplace, and 8.9 on sustainable tourism focusing on environmental sustainability. SDG 11 have targets 11.1 (adequate, safe, and affordable housing), 11.2 (sustainable transport), 11.3 (inclusive and sustainable urbanization), 11.4 (protecting and safeguarding cultural and natural heritages), 11.6 (environmental impact, air quality, waste management), and 11.7 (green and public spaces) focusing on environment. Targets 12.1 (sustainable consumption and production), 12.2 (sustainable management and use of natural resources), 12.4 (responsible management of chemicals and waste) and 12.5 (reduce waste generation) address environmental sustainability for SDG 12. All the targets of SDG 13, 14 and 15 are geared towards the achievement of environmental sustainability [37, 38].

In addition, 2019 data on expenditure on research and development, and scientific and technical journal articles were secured from the World Bank database. The study adopted the World Bank data on scientific and technical journal articles over the United Nations dataset because of the difference in databases/sources used for estimation. The UN data is gathered from Science-Metrix, which is operated and controlled by Elsevier and hence more biased toward their publications, but the World Bank uses the Scientific Information’s Science Citation Index (SCI) and Social Sciences Citation Index (SSCI) which is more open and includes over 12000 journals that are Scopus and Web of Science (WOS) indexed [39]. Furthermore, the World Bank data counts scientific and technical journal output per 1,000 inhabitants, whereas the UN data measures output per million people; thus, the World Bank data easily aids in highlighting research contributions for nations with smaller populations and outputs, while facilitating easy comparison among countries. The UN data on SDGs and World Bank scientific and technical journal output data were merged using a country code to create a single excel data file.

Furthermore, a spatial merged technique in ArcPro Software was employed to link the excel data file with a world country shapefile using the country codes. A data check and spatial exploration were undertaken to ensure the countries’ codes were consistent in each dataset with their corresponding spatial shape. Also, the data check was to look for missing data points, thus countries without scores. A spatial autofill based on the K-nearest-neighbour analysis (KNN) in ArcPro software was employed to fill in the missing data for countries without scores. The KNN assigned the mean score of 8 neighbouring countries for countries without scores. The descriptive analysis results indicated that SDG13 had the highest mean (M) of 81.53 and standard deviation (SD) of 17.33.

SDG 14 had the least mean of 60.45 and SD of 8.56. The average expenditure on research and development as a percentage of GDP was 0.78 with scientific and technical journal articles [per 1000 population] as 0.40. The highest expenditure on R&D was 4.5% of GDP with countries such as Israel and South Korea and the least amount was close to zero (0) percent with countries like Afghanistan, Benin, Comoros, Central Republic of Africa, etc. The mean amount spent by countries on R&D was 0.78% of their GDP (Table 1).

**Table 1:** Compiled dataset from the UN SDG database and the World Bank.

Variables	Minimum (Min)	Mean (M)	Maximum (Max)	Standard Deviation (SD)	Skewness	Kurtosis
[SDG 8]	37.47	72.86	91.85	9.47	-0.93	1.23
[SDG 11]	27.76	73.36	100.00	14.56	-0.97	0.38
[SDG 12]	17.77	74.82	96.21	16.23	-0.96	0.69
[SDG 13]	13.31	81.53	99.91	17.33	-1.48	2.83
[SDG 14]	5.80	60.45	83.07	8.56	-1.00	6.94
[SDG 15]	25.25	64.51	97.84	12.97	0.26	-0.12
Expenditure on research and development [% of GDP]	0.00	0.78	4.58	0.91	1.95	4.28
Scientific and technical journal articles [per 1,000 population]	0.00	0.40	2.51	0.59	1.73	1.98
Total Patent application	1.00	25742.81	1400661.00	108786.78	9.73	114.29
High-technology exports [% of manufactured exports]	0.00	12.04	62.25	9.83	1.60	4.00
Total Trademark Applications	151.00	31122.80	492729.00	52519.89	4.76	32.18

### Data analysis

The data analysis approaches adopted were spatial bivariate, spatial autocorrelation and geographic weighted regression (GWR). The spatial bivariate analysis was employed to assess the relationship between R&D expenditure on R&D outputs at the country level. Spatial bivariate analysis is based on local entropy which seeks to find a statistically significant relationship between two variables. The local entropy looked at the amount of randomness among countries and the ability to identify structural relationships (exponential, quadratic, sinusoidal, and even complex) between variables as against the linear relationship most convectional statistics generates [40].

The outcome from the spatial bivariate analysis were classified into six relationships, thus not significant-relationship between the entities is not statistically significant; the positive linear-dependent variable increased linearly with explanatory variable; negative relationship-dependent and independent variables decrease linearly; concave-dependent changes by a concave curve as explanatory increases; convex dependent changes by a convex curve as explanatory increases and undefined complex-significant relationship exist but the type of relationship is not visible and reliable [41]. A GWR model was used to assess the effect of expenditure on research and development [% of GDP], scientific and technical journal articles [per 1,000 population], total patent application, high-technology exports [% of manufactured exports] and total trademark applications as explanatory variables on SDGs [8, 11–15] as the dependant variables. The GWR equation is given as

$$y_{i}=\beta_{0}(u_{i}v_{i})+\sum_{k=1}^{p}\beta_{k}(u_{i},v_{j})x_{ik}+\varepsilon_{i}$$ (Eq 1)

Where:

Yi as the dependant variable (observation),

ui vi are the geographic locations of countries

K = 0,1…. p with p as the unknown geographic locations,

xik as the explanatory variables of countries and

εi as the residuals.

The study further sought to assess the reliability of the GWR model by assessing the randomness of the residuals using spatial autocorrelation analysis.

## Results

### Effect of R&D expenditure on R&D outputs

The relation between expenditure on R&D and scientific and technical journals articles produced concave relationships for Canada and Russia, 24 countries with convex relationships, 134 countries with positive relationships and 12 with undefined complex relationships (Appendix A in S1 File). Some of the countries which had the highest entropy were Denmark. Slovenia, Austria, Belarus, Lithuania, Estonia, and Germany with scores above 1.5, while Sao Tome. Cameroon, Kenya, Nigeria, Cuba, Jamaica, Haiti, Panama, El Salvador, Cayman Islands, Guatemala, Belize, Nicaragua and Honduras had values below 0.50. Expenditure on R&D and patent application produced entropy values from 1.18 to 0.19, with high entropy as North Korea (1.18), Japan (1.17), Brunei (1.15), Indonesia (1.15), Malaysia (1.15) and low entropy countries as Bermuda (0.19), Haiti (0.19), Jamaica (0.19), Cuba (0.19) (Appendix A in S1 File). About 10 countries (Argentina, Chile, Dominica, Grenada, Islas Malvinas, Martinique, St Lucia, St Vincent and Grenadines, Venezuela, and Uruguay) exhibited a concave relationship between R&D expenditure and patent. Countries which showed convex relationship were Samoa, Barbados, Bolivia, Brazil, Fiji, France Guiana, French Polynesia, Guyana, Jarvis Island, Mexico, Paraguay, Peru, Suriname, Tonga, Trinidad and Tobago, United States of America while over 84 countries had positive linear relationship between R&D expenditure and patent application. Entropy levels for expenditure on R&D and trademark application were from 0.18 to 0.34 with positive relationships between over 70 countries, 9 concave relationships, 23 convex relationships and 3 undefined relationships (Appendix A in S1 File).

**Fig 1:**
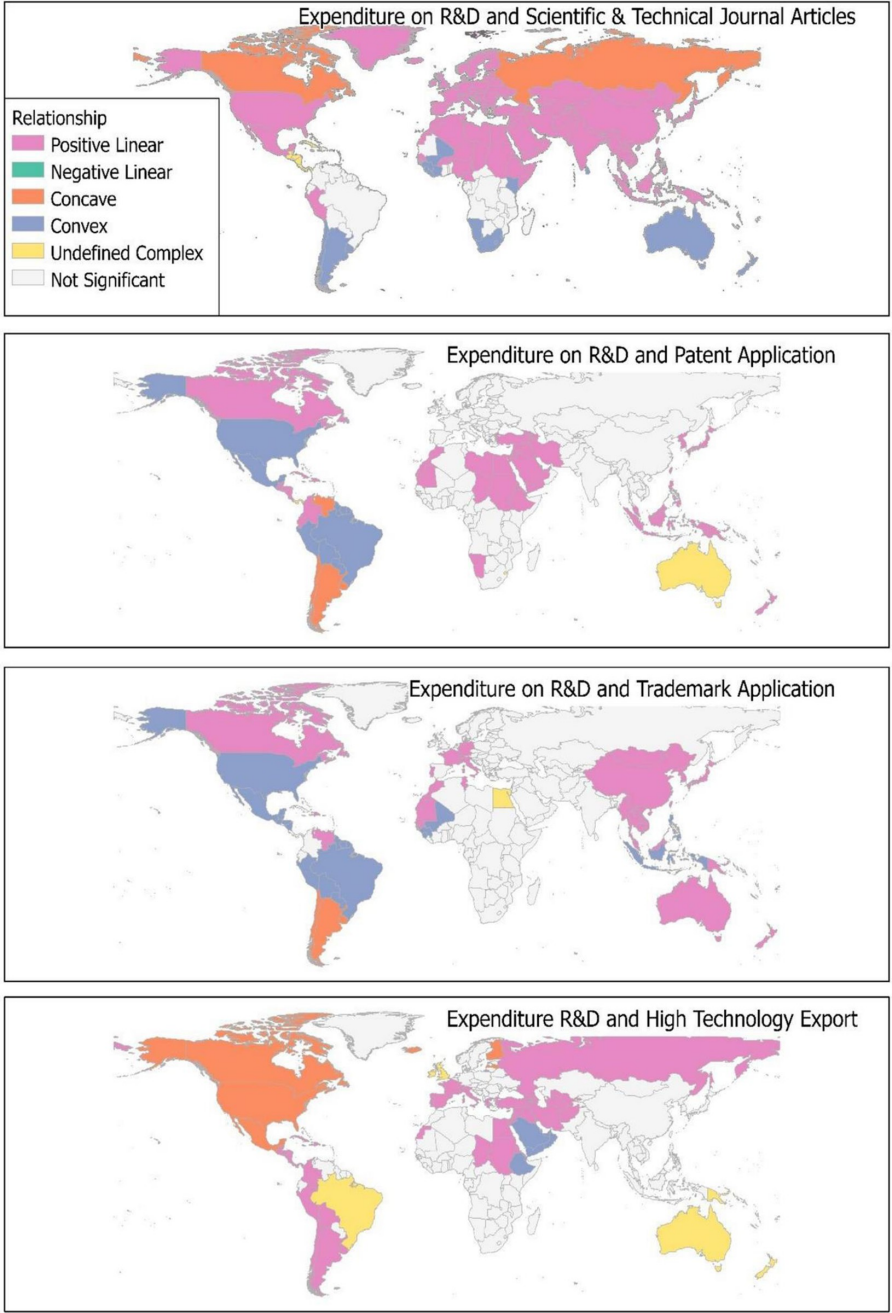
Local bivariate relationship between expenditure on R&D and R&D outputs. Source [42].

Entropy levels for countries per expenditure on R&D and high technology export were within the range of 1.34 to 0.52. Countries which showed a positive relationship between expenditure on R&D and high technology export were 62, 12 had convex relationships and 7 had concave relationships. Most countries did not have any significant relationship between expenditure on R&D and R&D outputs especially for countries in sub-Saharan Africa. Also, there was no significant relationship between expenditure on R&D, patent application and trademark application in most European countries (Fig 1).

### Effects of R&D expenditure on the environmentally sustainable development goals (ESDGs)

Geographic Weighted Regression was used to assess the effect of R&D on SDGs. R&D variables considered as explanatory variables were expenditure on research and development (% of GDP), scientific and technical journal articles (per 1,000 population), total patent application, total trademark applications, and high-technology exports (% of manufactured exports) as against SDGs as the dependant variable. GWR for SDG13 produced the highest R-square of 0.73 while the least was SDG 8 with an R-square of 0.62 (Table 2). Also, the model for SDG8 had the least AICC with a value of 1557.78 while SDG13 had the highest of 1792.05 (Table 2).

**Table 2:** GWR model diagnostics for R&D and SDGs.

SDG	R^2^	Adjusted R^2^	AICC	Sigma-Squared	Sigma-Squared MLE	Effective Degree of Freedom	Golden Search Rule (No. Neighbours)
SDG8	0.62	0.54	1557.78	40.95	34.28	195.06	75
SDG11	0.57	0.49	1783.80	107.94	90.30	194.93	89
SDG12	0.68	0.64	1742.37	94.16	82.94	205.22	126
SDG13	0.73	0.65	1792.05	104.52	81.16	180.93	61
SDG14	0.29	0.20	1630.26	58.62	52.17	207.36	136
SDG15	0.72	0.60	1705.96	66.78	47.92	167.21	46

The study assessed the reliability of the GWR models by checking for spatial clustering of the standardized residual from the models. The spatial autocorrelation test indicated a random effect for the standardized residuals signifying a good model for the GWR (Table 3).

**Table 3:** Spatial autocorrelation of standardized residual from GWR.

SDG	Moran’s Index	Expected Index	Variance	z-score	p-value	Relationship
SDG8	-0.0042	-0.0043	0.0006	0.00	0.99	Random
SDG11	0.0308	-0.0043	0.0006	1.39	0.16	Random
SDG12	0.0423	-0.0043	0.0006	1.88	0.06	Random
SDG13	0.0098	-0.0043	0.0006	0.56	0.56	Random
SDG14	0.0013	-0.0043	0.0006	0.23	0.82	Random
SDG15	0.0043	-0.0043	0.0006	0.34	0.73	Random

**Fig 2:**
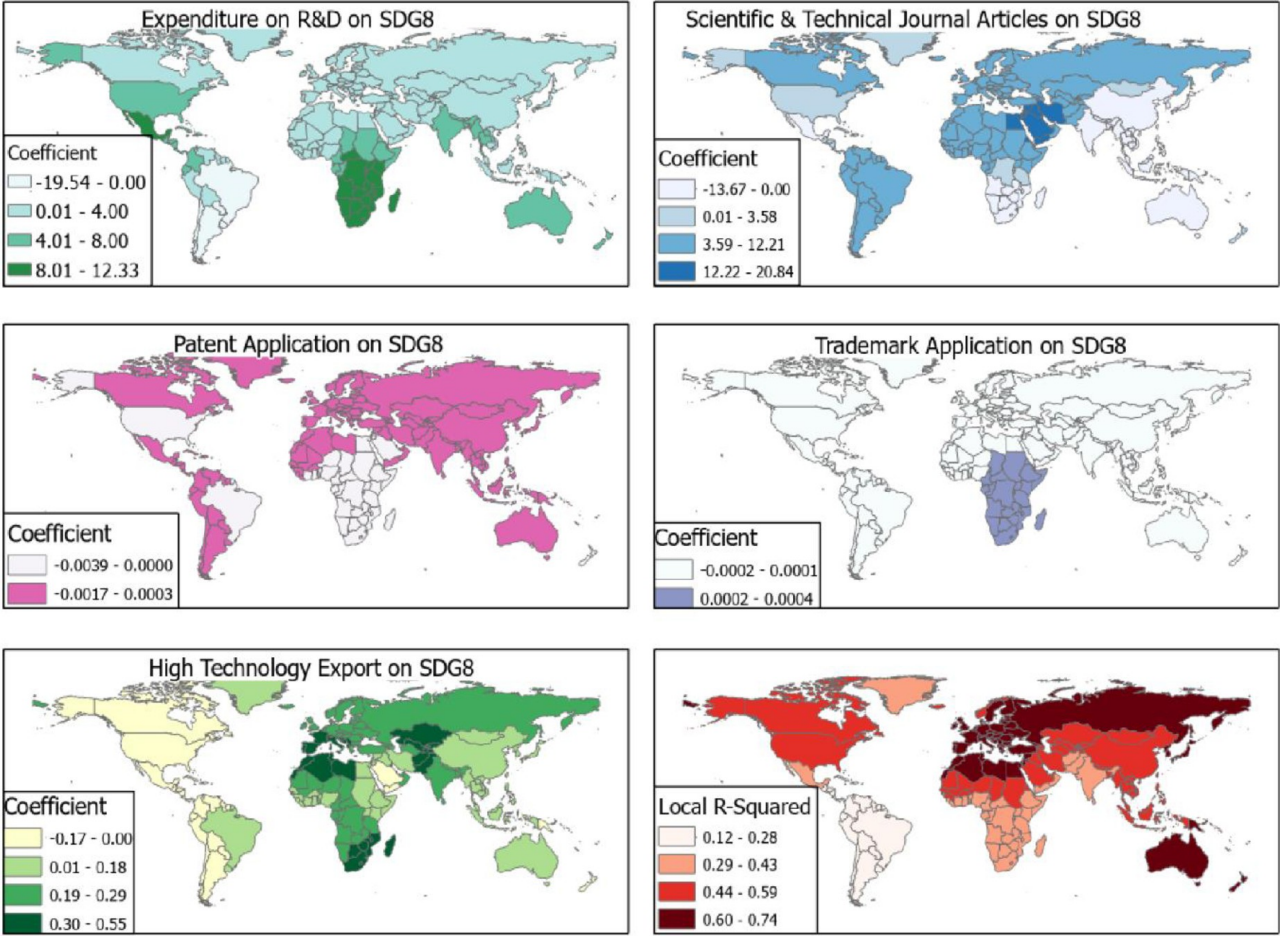
R&D and SDG 8. Source [42].

SDG 8 had the least Moran’s index of -0.0042, z-score = 0.00 and p = 0.99 while SDG 12 had the highest (Moran’s index = 0.0423, z-score = 1.88, p = 0.06). With the GWR model being fit, the study identified that scientific and technical journal articles [per 1,000 population] had the greatest effect on SDG 8 followed by expenditure on R&D and the least being patent application and trademark. The local R^2^ ranged from 0.12 to 0.74 with higher scores for European countries and Australia with R^2^ = 0.60–0.74 (Fig 2).

The effect of scientific and technical journal articles [per 1,000 population] on SDG 8 ranged from β = -13.67 to β = 20.44. Countries which experienced negative effects between scientific and technical journal articles [per 1,000 population], and SDG 8 were Australia, China, India, and Oceanic countries all with effects ranging from β = -13.67 to β = 0.00 (Fig 2). In the Middle East, countries like Egypt, Saudi Arabia, Jordan, Iraq and Iran had effects between β = 12.22 to β = 20.44. Southern African countries and Mexico showed a significant effect of expenditure on R&D on SDG 8 with β from 8.01 to 12.33. While there was negative (β = -19.54 to 0.00) effects between SDG 8 and expenditure on R&D for South American countries like Brazil, Argentina, Paraguay and Chile (Fig 2).

**Fig 3:**
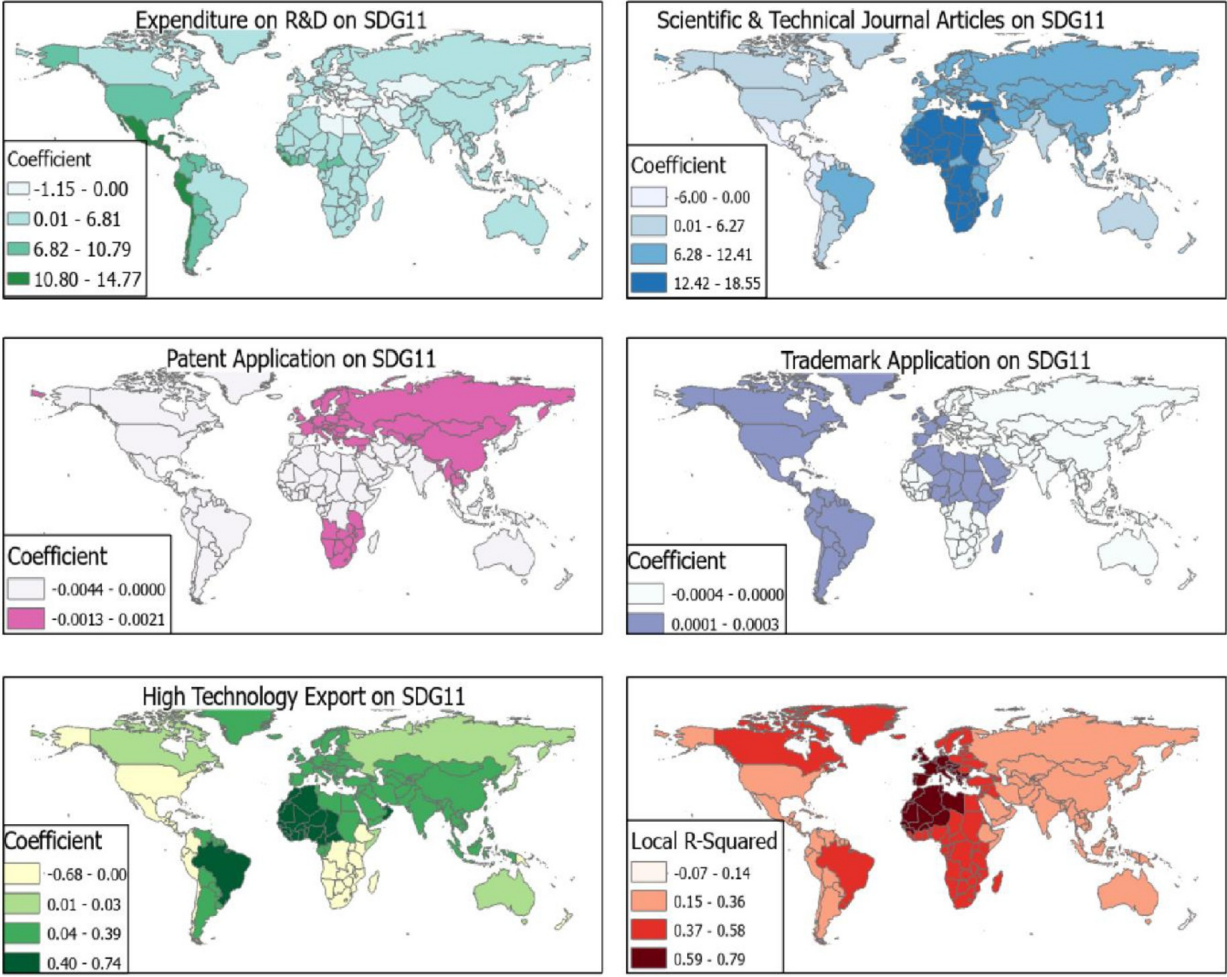
R&D and SDG 11. Source [42].

For SDG 11, the GWR model had R^2^ ranging from -0.07 to 0.79. R^2^ values were very high for African countries and European countries with scores within 0.59 to 0.79. Specifically, the effect of scientific and technical journal articles [per 1,000 population] was very high for most African countries with effects ranging from β = 12.42–18.55. Caribbean Islands experienced a negative effect between scientific and technical journal articles [per 1,000 population] and SDG 11 with β<0.00. However, expenditure on R&D had a profound effect on attainment of SDG 11 among countries in the Caribbean Islands, Mexico, Peru and Chile with β = 10.80–14.77 (Fig 3). Patent applications did not have any effect on SDG 11 in the North Americas but in Europe and Southern Africa (Fig 3).

**Fig 4:**
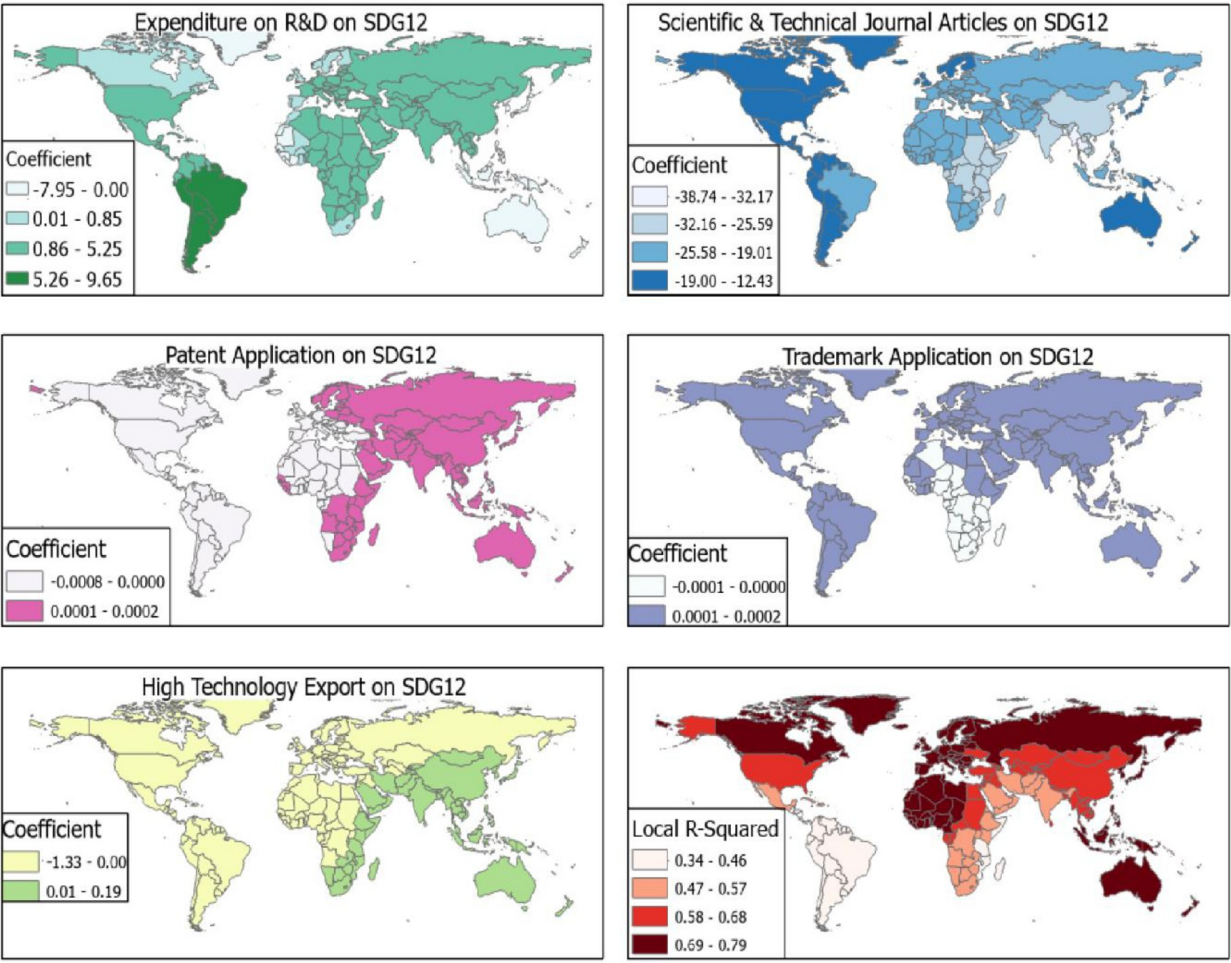
R&D and SDG 12. Source [42].

Scientific and technical journal articles [per 1,000 population] had a negative effect on SDG 12 for all the countries in the world with β = -38.74 to -12.43. Countries, where scientific and technical journal articles [per 1,000 population] had the most negative effects, were Central Africa, Madagascar, India, China, Nepal, Thailand, and Vietnam with β< -32.17 (Fig 4). The coefficient of expenditure on R&D on SDG 12 was high for South American countries (β = 5.26–9.65) and negative effects (β<0.00) for Australia, countries in the Ocean Islands and parts of West Africa thus, Mauritania, Senegal, Guinea, Sierra Leone and Liberia.

**Fig 5:**
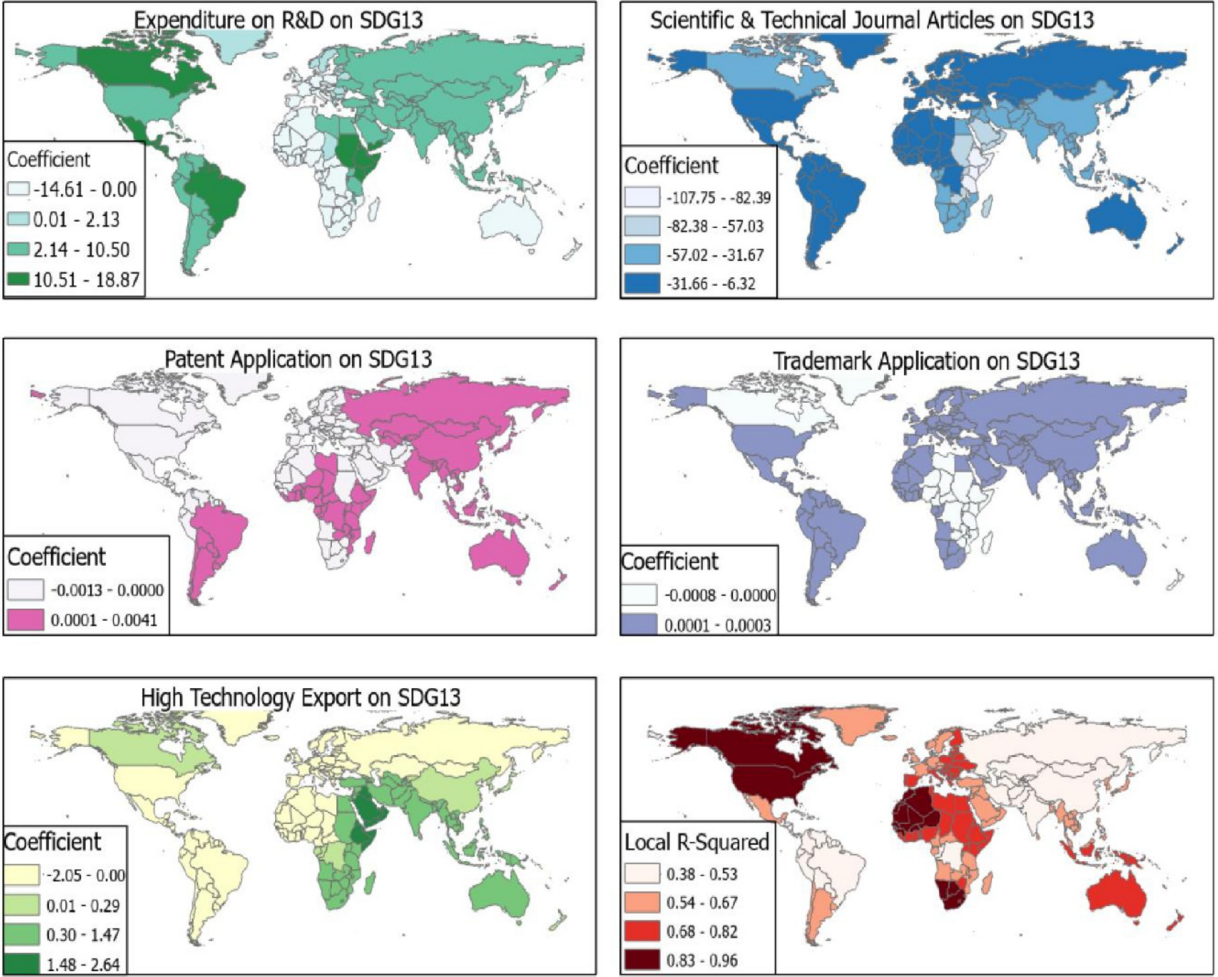
R&D and SDG 13. Source [42].

The effects of a patent application and trademark application on SDG 13 were very low compared with technology export especially for East Africa and Asian countries. Expenditure on R&D showed a significant positive effect on countries in the Horn of Africa, Canada and Brazil with β = 10.51–18.87 while having negative effects on West, Central and Southern African (β = -14.61–0.00). The R^2^ for the model was high for North America, and West and Southern Africa (R^2^ = 0.83–0.96) (Fig 5).

**Fig 6:**
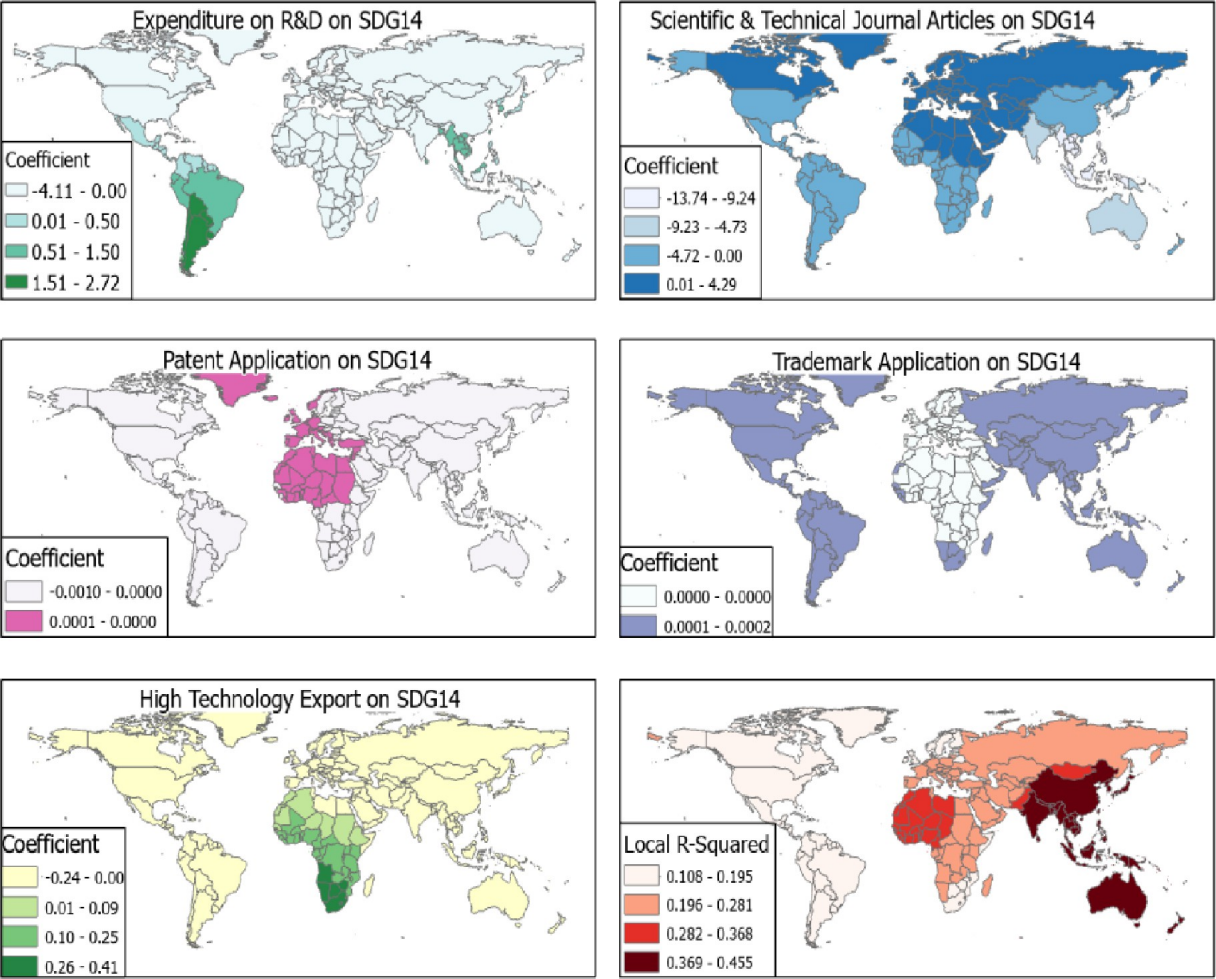
R&D and SDG 14. Source [42].

The effect of R&D on SDG 14 was low with all the independent variables producing β<5 and R^2^ < 0.5 (Fig 6).

**Fig 7:**
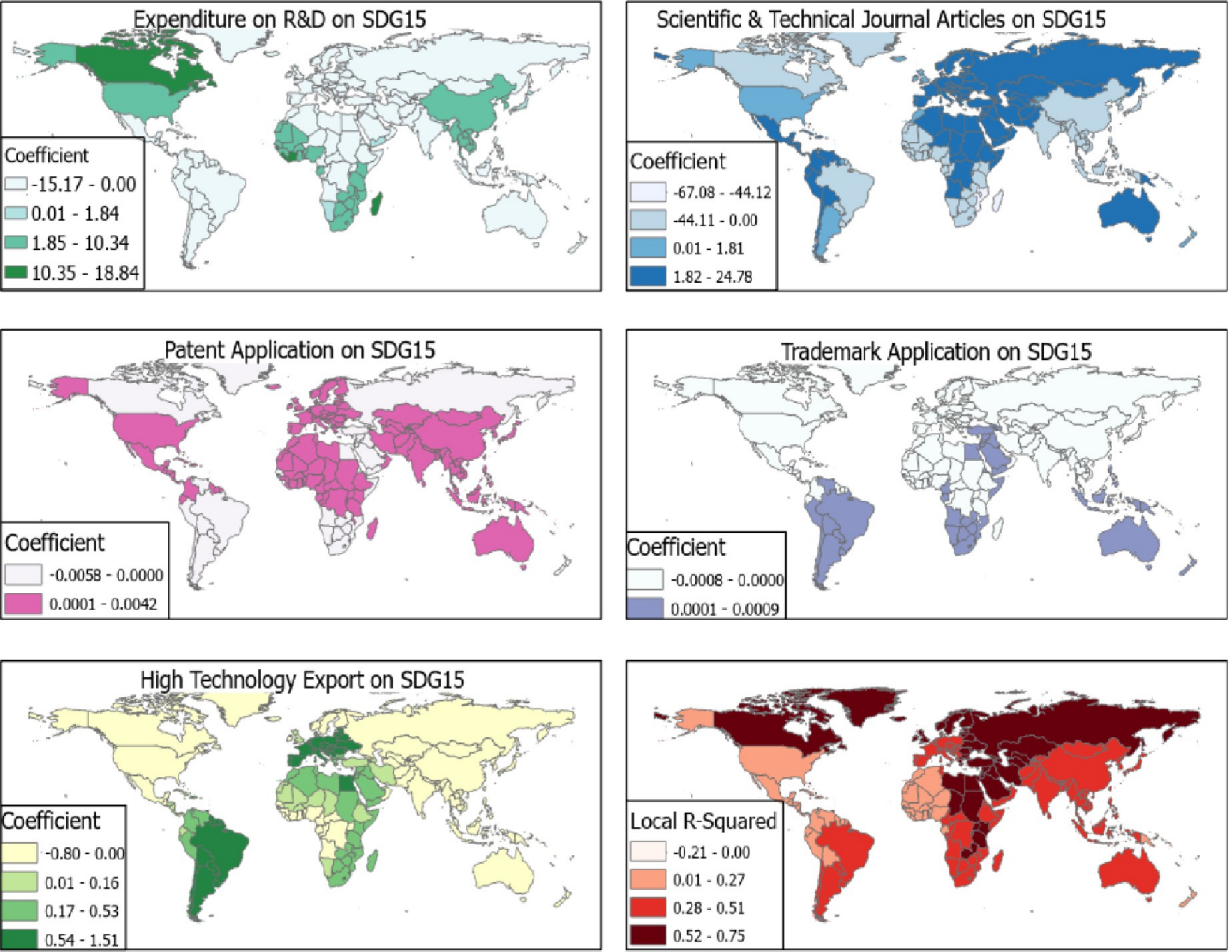
R&D AND SDG 15. Source [42].

On the contrary, the effect of R&D on SDG 15 was high for expenditure on R&D, scientific and technical journal articles [per 1,000 population] and high technology export. The effect of scientific and technical journal articles [per 1,000 population] on SDG 15 was positive for European countries with β = 1.48–24.78 (Fig 7). While the strong effect was identified between expenditure on R&D on SDG 15 in Canada, Cote d’ Ivoire and Liberia (β = 10.35–18.84). High technology export in Europe, South America and Egypt had positive effects on SDG 15 with β = 0.54–1.51.

## Discussion

R&D has been an efficient driving force in the development and growth of many countries around the world. The overall purpose of this study was to assess the contribution of R&D to the achievement of ESDGs. Spending on R&D is purported to generate a significant multiplier effect in most countries [10]. Therefore, our finding that there is a positive relationship between R&D expenditure and scientific and technical journal articles outputs among 134 countries reaffirm the concept of multiplier. The result supports the work of [9] who reported that there is a positive relationship between R&D investment and output in scientific and technical journal articles produced. By implication, as the expenditure on R&D rises, the output in terms of scientific and technical journal articles increases. This has an impact on other sectors, which will make use of the research output to drive new inventions and ideas.

Innovation is key to development, and as such expenditure on R&D is expected to boost patent right. In this study, R&D affecting the patent application in most countries is a necessity towards the achievement of the purpose of spending on R&D by most firms. The result suggests that so long as expenditure on R&D goes up the ability of most countries/firms seeking to have exclusive rights for innovations will increase. Thus, more inventions are made from the R&D expenditure to help solve societal problems; this has a direct effect on the achievement of the ESDGs. The result is in line with the findings of [43] that intellectual property protection is important in development, and hence countries must standardize their inventions and innovations for scientific acceptability as they seek exclusive rights to their inventions. This is also re-echoed by [44, 45] who found a significant relationship between R&D expenditure and patent application. The authors further explained that inventors are motivated to do more when they have the exclusive right to their inventions.

Even though R&D offers better opportunities for innovations, knowledge, and novelty in human life [1]. Expenditure on R&D is expected to be proportional to its output [5, 6] as has been observed that countries with more expenses on research tend to reap higher returns [6, 8]. However, R&D expenditure doesn’t always produce similar effects on developments [15], as the study found negative relationship between R&D expenditure and SDG 8 among countries in South America, Australia, China, India, Oceania and the Middle East. Furthermore, [46] found negative effects between R&D expenditure and economic growth among several countries, globally. Zhu et al., [9] argue that when R&D expenditure is highly financed by government the effect on companies and firm’s growth tends to be lower as they are guided by governmental pressure. Contrary, [16] found positive spill over effects of defence R&D by government on the private sector.

The study found that scientific and technical journal articles played significant role in the achievement of ESDGs in Africa. R&D expenditure adds value to the achievement of SDG 11 [47]. It is however not surprising that most researchers in the Latin America/Caribbean prioritize SDG 11 since it is related to sustainable cities in relation to climate change. However, the fact that scientific and technical journal articles produce a negative effect on environmental sustainability in some countries/regions is disturbing. This perhaps might be because research outputs in these regions are not being effectively utilized especially in policy implementation as policy makers rely on their intellects and experience instead [48].

Moreover, SDG 13 talks about taking urgent action to combat climate change and its impacts. Achieving SDG 13 is highly dependent on research which is influenced by the expenditure on R&D [49]. Observing a significant effect of R&D expenditure on research output among Africa and North America countries is an indication of the fact that research is a necessity in achieving SDG 13 and hence its consequential impact on the sustainability of the environment. This is in tandem with the assertion of [50] that many countries recognise climate change as one of the serious challenges of the 21^st^ century and strives towards resilience and adaptation programmes. This doesn’t take away the fact that Africans’ expenditure towards R&D is small. Africa is highly vulnerable to climate change; hence it is only prudent that more resources are committed to SDG 13 research. Also, it is no surprising that more than half of the researchers in African focus on climate change studies although they have come from different research backgrounds [47]. It is important governments and international organisations in Africa dedicate more funds to researchers to study the effects and possible solutions to climate change.

## Conclusions

Having a sustainable world is not only about the growth and development of economies but also the environment; an aspect that is keenly concentrated in the SDGs of the United Nations. Yet, the inequality in R&D expenditures of countries and continents coupled with the unequal outputs from R&D to the growth and development of nations is important in achieving the SDGs. We sought to fill the research gap on limited research on the effects of R&D expenditure and R&D outputs on ESDGs on one hand and R&D expenditure on R&D outputs on another hand. The result of this study is hoped to go a long way in advocating for commitment in both governmental and non-governmental R&D funding across the globe as well as funds distribution towards ESDGs. Following the result, we observed that R&D expenditure affects R&D outputs and achievement of ESGs. So, based on the *H1* that R&D expenditure has no effects on R&D outputs (scientific and journals technical articles, total patent application, total trademark application and high technology export), we reject the null hypothesis and conclude that there is no significant evidence to support the claim and hence accept the alternate hypothesis.

Lastly, regarding R&D outputs on ESDGs, it is certain from this study that R&D expenditure is an advocate in affecting the achievement of SDGs 8 and 11 in all countries irrespective of the level of development. Therefore, on the bases of the *H2* that R&D expenditure has no significant effects on the attainment of the SDGs, we reject the null hypothesis because there is not enough evidence to support the claim and rather accept the alternate hypothesis. The study, therefore, recommends that R&D expenditure be boosted in countries, especially on the African continent since the multiplier impact extends beyond economic development to environmental sustainability which is necessary for the continent to abate the challenges of climate change.

## Supporting information

S1 File (ZIP)

## References

[1] Ho L.; Alonso A.; Forio M.A.E.; Vanclooster M.; Goethals P.L.M. Water research in support of the Sustainable Development Goal 6: A case study in Belgium. J. Clean. Prod. 2020, 277, 10.1016/j.jclepro.2020.124082

[2] Lurie N.; Keusch G. T.; Dzau V. J. Urgent lessons from COVID 19: Why the world needs a standing, coordinated system and sustainable financing for global research and development. *Lancet* 2021, 397(10280), 1229–1236. doi: 10.1016/S0140-6736(21)00503-1 33711296 PMC7993931

[3] Picone F.; Buonocore E.; Chemello R.; Russo G.F.; Franzese P.P. Exploring the development of scientific research on marine protected areas: From conservation to global ocean sustainability. *Ecol*. *Inform*. 2020, 61, 1–8.

[4] Cazavan-jeny A.; Jeanjean T. The negative impact of R&D capitalization: A value relevance approach. *Europ*. *Account*. *Rev*. 2007, 15(1), 37–61. 10.1080/09638180500510384

[5] Akcali B.Y.; Sismanoglu E. Innovation and the effect of research and development (R&D) expenditure on growth in some developing and developed countries. *Proc*. *Soc*. *and Behav*. *Sci*. 2015, 195, 768–775. 10.1016/j.sbspro.2015.06.474

[6] Gumus E.; Celikay F. R&D Expenditure and economic growth: New empirical evidence. *Margin*: *J*. *Appl*. *Econ*. *Res*. 2015, 9(3), 205–217. 10.1177/0973801015579753

[7] UNESCO Institute for Statistics [UIS]. UIS releases new data for SDG 9.5 on research and development, http://uis.unesco.org/en/news/uis-releases-new-data-sdg-9-5-research-and-development (accessed on 22th July, 2022)

[8] Gülmez A.; Yardmcolu F. OECD Ülkelerinde Ar-Ge Harcamalar ve Ekonomik Büyüme likisi: Panel Ebütünleme ve Panel Nedensellik Analizi (1990–2010), *Mal*. *Derg*. 2012, 163, 335–353.

[9] Zhu H.; Zhao S; Abbas A. Relationship between R&D grants, R&D investment, and innovation performance: The moderating effect of absorptive capacity, *J*. *Pub*. *Aff*. 2019, e1973–. 10.1002/pa.1973

[10] Roe, A. R.; Round, J. Framework: the channels for indirect impacts. WIDER Working Paper 2017/79, https://www.wider.unu.edu/sites/default/files/wp2017-79.pdf (accessed 22nd July, 2022)

[11] Spilimbergo, A.; Symansky, S.; Schindler, M. Fiscal multipliers: IMF staff position note SPN/09/11 (Washington: International Monetary Fund) 2009. https://www.imf.org/external/pubs/ft/spn/2009/spn0911.pdf

[12] Sloboda B.W; Yao V.W. Interstate spillovers of private capital and public spending. *Ann*. *Reg*. *Sci*. 2008, 42(3), 505–518

[13] Prentice C.; Wange X.; Manhas P. S. The spillover effect of airport service experience on destination revisit intention. *J*. *Hosp*. *and Tour*. *Manag*. 2021, 48, 119–127, 10.1016/j.jhtm.2021.06.001

[14] Cheung K.; Ping L. Spillover effects of FDI on innovation in China: Evidence from the provincial data. *China Econ*. *Rev*. 2004, 15, 25–44. 10.1016/S1043-951X(03)00027-0

[15] Domański B. & Gwosdz K. Multiplier effects in local and regional development. *Quaes*. *Geogr*. 2010, 29 *(*2*)*, 27–37. 10.2478/v10117-010-0012-7

[16] Moretti E.; Steinwender C.; Reenen J. V. The intellectual spoils of War? Defence R&D, Productivity and International Spill overs 2021. https://eml.berkeley.edu/~moretti/military.pdf

[17] Arrow K. J. The economic implications of learning by doing. *Rev*. *Econ*. *Stud*. 1962, 29(3), 155–173. 10.2307/2295952

[18] Carillo, M. R.; Papagni, E. *Scientific research*, *externalities and economic growth* [Discussion Papers]. Department of Economic Studies, University of Naples, Italy Discussion Papers 2006. https://ideas.repec.org/p/prt/dpaper/11_2006.html

[19] Ildırar M.; Özmen M.; İşcan E. The effect of research and development expenditures on economic growth: new evidences. *Inter*. *Conf*. *Euras*. *Econo*. 2016. 10.36880/C07.01776

[20] Tuna K.; Kayacan E.; Bekta H. The relationship between research & development expenditures and economic growth: The Case of Turkey. World Conference on Technology, Innovation and Entrepreneurship. *Proc*. *Soc*. *and Behav*. *Sci*. 2015, 195, 501–507, doi: 10.1016/j.sbspro.2015.06.255

[21] Silaghi M. I. P.; Alexa D.; Jude C.; Litan C. Do business and public sector research and development expenditures contribute to economic growth in Central and Eastern European Countries? A dynamic panel estimation. *Econ*. *Model*. 2013, 36, 108–119. 10.1016/j.econmod.2013.08.035

[22] Surani S.; Gendron W.; Maredia S. The Economic Impact of Research and Development. *Econometrics*, 2017 3161

[23] Blanco L. R.; Gu J.; Prieger J. E. The impact of research and development on economic growth and productivity in the U.S. States. *Southern Econ*. *Jour*. 2017, 82(3), 914–934. 10.1002/soej. 12107

[24] Zhou L.; Xia L. How R&D investments influence TFP growth: Evidence from China’s large and medium sized industrial enterprises, *Front*. *Econ*. *China*, 2010, 5(4), 537–558.

[25] Inekwe J. N. The contribution of R&D expenditure to economic growth in developing countries. *Soc*. *Indica*. *Res*. 2014, 14(1), 1–19.

[26] Özcan B.; Ar A. Aratrma-Gelitirme Harcamalar ve Ekonomik Büyüme likisi: Panel Veri Analizi, *Mal*.*Derg*. 2014, 166, 39–55.

[27] Gülolu B.; Tekin R. B. A panel causality analysis of the relationship among research and development, innovation and economic growth in high-income OECD countries. *Euras*. *Econ*. *Rev*. 2012, 2(1), 32–47.

[28] Gross, D. P.; Sampat, B. N. Inventing the endless frontier: the effects of the World War II research effort on post-war innovation. NBER Working Paper Series, Working Paper 27375 http://www.nber.org/papers/w27375 (accessed on 20^th^ June, 2022)

[29] Szmigiera, M. Total global R&D spending 1996–2018. https://www.statista.com/statistics/1105959/total-research-and-development-spending-worldwide-ppp-usd/ (accessed on 22^nd^ July, 2021).

[30] Congressional Research Service [CRS]. Global research and development expenditures: Fact Sheet. https://crsreports.congress.govR44283 version 14 updated (accessed 15^th^ May, 2022)

[31] UNESCO Institute for Statistics [UIS]. Global investments in R & D. Fact sheet No 59, FS/2020/SCI/59. http://uis.unesco.org (accessed on 20^th^ May, 2022)

[32] Simpkin V.; Namubiru-Mwaura E.; Clarke L.; Mossialos E. Investing in health R&D: where we are, what limits us, and how to make progress in Africa. *BMJ Glob*. *Health* 2019, 4: e001047. doi: 10.1136/bmjgh-2018-001047 PMC6407556 30899571

[33] R&D Magazine. Global R&D funding forecast. https://www.rdmag.com/article/2016/02/2016-global-rd-funding-forecast-0 (3^rd^ April, 2022)

[34] UNESCO Institute for statistics, [UIS]. Database. http://uis.unesco.org (accessed on 20^th^ May, 2022)

[35] Mikulic, M. Total global pharmaceutical R&D spending 2012–2026. Statista. https://www.statista.com/statistics/309466/global-r-and-d-expenditure-for-pharmaceuticals/ (accessed on 27^th^ August, 2022).

[36] International Labour Organisation. Relevant SDG targets related to environment and green jobs. https://www.ilo.org/global/topics/dw4sd/themes/green-jobs/WCMS_558559/lang—en/index.htm (accessed on 14^th^ May, 2022)

[37] Elder M.; Olsen S. H. The design of environmental priorities in the SDGs. *Global Policy*. 2019; 10:70–82.

[38] Arora N. K.; Mishra I. United Nations Sustainable Development Goals 2030 and environmental sustainability: race against time. *Environmental Sustainability*. 2019 2(4), 339–42.

[39] World Bank. Scientific and technical journals. https://data.worldbank.org/indicator/IP.JRN.ARTC.SC (accessed on 11th August 2023)

[40] Guo D. Local entropy map: A nonparametric approach to detecting spatially varying multivariate relationships. *Inter*. *J*. *Geographical Inform*. *Sci*. 2010, 24(9), 1367–1389. 10.1080/13658811003619143

[41] ESRI. Local bivariate relationships (Spatial statistics). https://pro.arcgis.com/en/pro-app/latest/tool-reference/spatial-statistics/localbivariaterelationships.htm. (Accessed on 14^th^ May, 2022)

[42] Environment and Geospatial Sciences Lab (2023). Maps for Research and Development (R&D) and Sustainable Development Goals (SDGs). Department of Geography Education: University of Education, Winneba

[43] Hussinger K.; Schwiebacher F. The value of disclosing IPR to open standard setting organizations. *Ind*. *and Innov*. 2015, 22(4), 321–344.

[44] Das R.C. Interplays among R&D spending, patent and income growth: new empirical evidence from the panel of countries and groups. *J*. *Innov*. *Entrep*. 2020, 9(8). 10.1186/s13731-020-00130-8

[45] Prodan I. Influence of research and development expenditures on number of patent applications: selected case studies in OECD countries and central Europe, 1981–2001. *Appl*. *Econometrics and Inter*. *Devel*. 2005, 5(4) https://www.usc.es/economet/journals1/aeid/aeid541.pdf

[46] Lin Y.; Dong D.; Wang J. The negative impact of uncertainty on R&D investment: International evidence. *Sustainability* 2021, 13, 2746. 10.3390/su13052746

[47] Salvia A. L.; Filho W. L.; Brandli L. L.; Griebeler J. S. Assessing research trends related to sustainable development Goals: Local and global issues. *J*. *Clean*. *Prod*. 2019, 08:841–9. 10.1016/j.jclepro.2018.09.242

[48] Harris R. The impact of research on development policy and practice: This much we know. In: Impact of information society research in the global south, Chib A., May J., Barrantes R. Eds. Springer, Singapore 2015; 10.1007/978-981-287-381-1_2

[49] Caiado R.G.G.; Leal F W.; Quelhas O.L.G.; de Mattos Nascimento D.L.; Ávila L.V. A literature-based review on potentials and constraints in the implementation of the Sustainable Development Goals. *J*. *Clean*. *Prod*. 2018, 198, 1276–1288.

[50] Verner G.; Schutte S.; Knop J.; Sankoh O.; Sauerborn R. Health in climate Change research from 1990 to 2014: Positive trend, but still underperforming. *Glob*. *Health Act*. 2016, 9, 30723 doi: 10.3402/gha.v9.30723 27339855 PMC4917601

