# Supplementary material for: Global outlook of the multiplier effect of research and development on environmental sustainability
Source: PLoS One. 2023 Sep 21;18(9):e0291370. doi: 10.1371/journal.pone.0291370 (PMC10513248; doi:10.1371/journal.pone.0291370)
Supplement: S1 File — (ZIP) [file pone.0291370.s001.zip › SI Appendices.docx]

**Appendix A**

**Table A1:** **Geographic weighted regression of expenditure R& D and scientific research outputs**

| Country | Entropy | Intercept | Polynomial Intercept | Coefficient | Polynomial Coefficient  (Linear) | Polynomial Coefficient (Square) | P-Value | R2 | Polynomial  R2 | Relationship |
| --- | --- | --- | --- | --- | --- | --- | --- | --- | --- | --- |
| **Expenditure on R&D (% of GDP) and Scientific and technical journal articles (per 1,000 population)** | | | | | | | | | | |
| Ethiopia | 0.77 | 0.00 | 0.01 | 0.58 | 0.35 | 0.22 | 0.01 | 0.93 | 0.93 | Positive Linear |
| Heard Island & McDonald Islands | 0.83 | -0.09 | -0.01 | 1.90 | 0.28 | 3.38 | 0.01 | 0.90 | 0.94 | Convex |
| Canada | 1.15 | 0.02 | -0.03 | 1.35 | 2.37 | -1.69 | 0.01 | 0.89 | 0.92 | Concave |
| Oman | 0.90 | 0.02 | 0.04 | 0.54 | 0.17 | 0.37 | 0.01 | 0.86 | 0.88 | Positive Linear |
| Afghanistan | 0.93 | 0.02 | 0.04 | 0.54 | 0.20 | 0.34 | 0.01 | 0.86 | 0.88 | Positive Linear |
| French Southern & Antarctic Lands | 0.78 | -0.09 | 0.00 | 1.91 | 0.02 | 4.35 | 0.01 | 0.86 | 0.95 | Convex |
| Chad | 0.74 | -0.01 | -0.02 | 0.59 | 0.72 | -0.12 | 0.01 | 0.85 | 0.85 | Positive Linear |
| Djibouti | 0.89 | 0.01 | 0.03 | 0.54 | 0.14 | 0.40 | 0.01 | 0.85 | 0.87 | Positive Linear |
| Bouvet Island | 0.74 | -0.09 | 0.00 | 1.79 | 0.17 | 3.77 | 0.01 | 0.83 | 0.93 | Convex |
| Sudan | 0.83 | 0.00 | 0.02 | 0.56 | 0.26 | 0.30 | 0.01 | 0.81 | 0.81 | Positive Linear |
| United States | 0.86 | 0.00 | -0.01 | 1.26 | 1.53 | -0.51 | 0.01 | 0.78 | 0.78 | Positive Linear |
| Mongolia | 1.00 | 0.01 | 0.01 | 0.49 | 0.50 | -0.02 | 0.01 | 0.77 | 0.77 | Positive Linear |
| Iran | 0.97 | 0.04 | 0.04 | 0.53 | 0.40 | 0.13 | 0.01 | 0.77 | 0.76 | Positive Linear |
| Turkmenistan | 1.02 | 0.04 | 0.05 | 0.53 | 0.34 | 0.18 | 0.01 | 0.76 | 0.76 | Positive Linear |
| Uzbekistan | 1.02 | 0.04 | 0.05 | 0.53 | 0.34 | 0.18 | 0.01 | 0.76 | 0.76 | Positive Linear |
| Namibia | 0.63 | -0.08 | 0.02 | 1.62 | -0.58 | 5.29 | 0.01 | 0.76 | 0.99 | Convex |
| Lesotho | 0.71 | -0.08 | 0.01 | 1.63 | -0.43 | 4.97 | 0.01 | 0.76 | 0.97 | Convex |
| Bahrain | 0.98 | 0.03 | 0.04 | 0.53 | 0.37 | 0.16 | 0.01 | 0.76 | 0.76 | Positive Linear |
| Qatar | 0.98 | 0.03 | 0.04 | 0.53 | 0.37 | 0.16 | 0.01 | 0.76 | 0.76 | Positive Linear |
| United Arab Emirates | 0.98 | 0.03 | 0.04 | 0.53 | 0.37 | 0.16 | 0.01 | 0.76 | 0.76 | Positive Linear |
| South Africa | 0.67 | -0.08 | 0.02 | 1.62 | -0.56 | 5.24 | 0.02 | 0.76 | 0.99 | Convex |
| Saudi Arabia | 0.98 | 0.03 | 0.04 | 0.53 | 0.35 | 0.18 | 0.01 | 0.76 | 0.76 | Positive Linear |
| Kuwait | 1.00 | 0.04 | 0.05 | 0.53 | 0.39 | 0.14 | 0.01 | 0.76 | 0.75 | Positive Linear |
| Yemen | 0.97 | 0.03 | 0.05 | 0.53 | 0.26 | 0.27 | 0.01 | 0.75 | 0.75 | Positive Linear |
| Mexico | 0.70 | 0.01 | -0.02 | 1.03 | 1.93 | -1.58 | 0.01 | 0.74 | 0.78 | Positive Linear |
| Eritrea | 0.96 | 0.02 | 0.04 | 0.54 | 0.24 | 0.30 | 0.01 | 0.74 | 0.74 | Positive Linear |
| Cocos (Keeling) Islands | 0.93 | -0.06 | 0.04 | 1.55 | -0.25 | 4.17 | 0.01 | 0.74 | 0.86 | Convex |
| Swaziland | 0.67 | -0.08 | 0.02 | 1.56 | -0.58 | 5.27 | 0.01 | 0.73 | 0.98 | Convex |
| Chile | 0.80 | -0.02 | 0.06 | 1.50 | -0.56 | 4.89 | 0.01 | 0.73 | 0.87 | Convex |
| Iraq | 1.06 | 0.06 | 0.05 | 0.52 | 0.62 | -0.10 | 0.01 | 0.72 | 0.71 | Positive Linear |
| Azerbaijan | 1.10 | 0.06 | 0.05 | 0.52 | 0.59 | -0.07 | 0.01 | 0.71 | 0.70 | Positive Linear |
| Byelarus | 1.52 | 0.05 | -0.02 | 1.09 | 1.68 | -0.76 | 0.01 | 0.71 | 0.71 | Positive Linear |
| Lithuania | 1.52 | 0.05 | -0.02 | 1.09 | 1.68 | -0.76 | 0.01 | 0.71 | 0.71 | Positive Linear |
| Armenia | 1.13 | 0.06 | 0.06 | 0.52 | 0.58 | -0.07 | 0.01 | 0.71 | 0.70 | Positive Linear |
| Jordan | 1.12 | 0.06 | 0.06 | 0.52 | 0.56 | -0.05 | 0.01 | 0.71 | 0.70 | Positive Linear |
| Syria | 1.16 | 0.07 | 0.06 | 0.51 | 0.57 | -0.06 | 0.01 | 0.71 | 0.70 | Positive Linear |
| Falkland Islands (Islas Malvinas) | 0.84 | -0.03 | 0.06 | 1.44 | -0.66 | 5.07 | 0.02 | 0.70 | 0.87 | Convex |
| Pakistan | 0.95 | 0.02 | 0.04 | 0.50 | 0.16 | 0.37 | 0.01 | 0.70 | 0.72 | Positive Linear |
| British Indian Ocean Territory | 0.78 | -0.06 | 0.02 | 1.25 | -0.31 | 3.80 | 0.01 | 0.69 | 0.90 | Convex |
| Argentina | 0.81 | -0.01 | 0.07 | 1.47 | -0.78 | 5.29 | 0.02 | 0.69 | 0.85 | Convex |
| Latvia | 1.52 | 0.06 | -0.02 | 1.08 | 1.73 | -0.83 | 0.01 | 0.69 | 0.69 | Positive Linear |
| Uruguay | 0.81 | -0.01 | 0.08 | 1.46 | -0.83 | 5.40 | 0.01 | 0.69 | 0.85 | Convex |
| Estonia | 1.52 | 0.07 | -0.03 | 1.09 | 1.80 | -0.90 | 0.01 | 0.68 | 0.68 | Positive Linear |
| Georgia | 1.15 | 0.07 | 0.05 | 0.52 | 0.84 | -0.32 | 0.01 | 0.68 | 0.68 | Positive Linear |
| Finland | 1.47 | 0.10 | -0.09 | 1.09 | 2.30 | -1.51 | 0.01 | 0.67 | 0.71 | Positive Linear |
| Egypt | 1.07 | 0.06 | 0.04 | 0.52 | 0.78 | -0.25 | 0.01 | 0.66 | 0.66 | Positive Linear |
| Moldova | 1.36 | 0.06 | 0.02 | 0.96 | 1.33 | -0.52 | 0.01 | 0.66 | 0.66 | Positive Linear |
| Guinea | 0.69 | -0.05 | 0.01 | 1.35 | -0.81 | 8.34 | 0.01 | 0.66 | 0.86 | Convex |
| Sierra Leone | 0.69 | -0.05 | 0.01 | 1.35 | -0.81 | 8.34 | 0.01 | 0.66 | 0.86 | Convex |
| West Bank | 1.12 | 0.08 | 0.06 | 0.51 | 0.66 | -0.15 | 0.01 | 0.65 | 0.64 | Positive Linear |
| Gaza Strip | 1.13 | 0.07 | 0.06 | 0.51 | 0.68 | -0.16 | 0.01 | 0.65 | 0.64 | Positive Linear |
| Israel | 1.13 | 0.07 | 0.06 | 0.51 | 0.68 | -0.16 | 0.01 | 0.65 | 0.64 | Positive Linear |
| Lebanon | 1.16 | 0.08 | 0.07 | 0.50 | 0.64 | -0.13 | 0.01 | 0.65 | 0.64 | Positive Linear |
| Maldives | 0.84 | -0.03 | 0.03 | 1.23 | -0.22 | 3.54 | 0.01 | 0.65 | 0.80 | Convex |
| Poland | 1.51 | 0.10 | 0.11 | 0.97 | 0.88 | 0.11 | 0.01 | 0.64 | 0.63 | Positive Linear |
| Ukraine | 1.34 | 0.06 | 0.00 | 0.96 | 1.52 | -0.81 | 0.01 | 0.63 | 0.64 | Positive Linear |
| Norway | 1.43 | 0.13 | -0.04 | 1.02 | 2.13 | -1.37 | 0.01 | 0.61 | 0.65 | Positive Linear |
| Sweden | 1.43 | 0.13 | -0.04 | 1.02 | 2.13 | -1.37 | 0.01 | 0.61 | 0.65 | Positive Linear |
| Midway Islands | 1.04 | -0.06 | 0.01 | 0.54 | 0.04 | 0.53 | 0.01 | 0.61 | 0.67 | Convex |
| Russia | 1.32 | 0.03 | -0.05 | 0.83 | 1.88 | -1.30 | 0.01 | 0.60 | 0.70 | Concave |
| Slovakia | 1.47 | 0.10 | 0.10 | 0.96 | 0.93 | 0.04 | 0.01 | 0.60 | 0.59 | Positive Linear |
| Cyprus | 1.13 | 0.09 | 0.05 | 0.51 | 0.89 | -0.37 | 0.01 | 0.60 | 0.61 | Positive Linear |
| Czech Republic | 1.50 | 0.10 | 0.11 | 0.94 | 0.80 | 0.19 | 0.01 | 0.60 | 0.58 | Positive Linear |
| Hungary | 1.50 | 0.10 | 0.12 | 0.94 | 0.80 | 0.18 | 0.01 | 0.59 | 0.58 | Positive Linear |
| Kazakhstan | 1.10 | 0.01 | 0.04 | 0.91 | 0.50 | 0.82 | 0.01 | 0.59 | 0.59 | Positive Linear |
| Mali | 0.76 | -0.05 | 0.01 | 1.41 | -0.34 | 6.75 | 0.01 | 0.58 | 0.70 | Convex |
| Sri Lanka | 0.93 | -0.01 | 0.05 | 1.14 | -0.32 | 3.65 | 0.01 | 0.58 | 0.74 | Convex |
| Liberia | 0.66 | -0.04 | 0.01 | 1.17 | -0.76 | 7.70 | 0.01 | 0.58 | 0.83 | Convex |
| China | 1.12 | 0.01 | -0.01 | 0.60 | 0.93 | -0.40 | 0.01 | 0.57 | 0.58 | Positive Linear |
| Myanmar (Burma) | 1.06 | 0.01 | -0.02 | 0.65 | 1.06 | -0.48 | 0.01 | 0.55 | 0.57 | Positive Linear |
| Thailand | 1.04 | 0.01 | -0.02 | 0.65 | 1.05 | -0.48 | 0.01 | 0.55 | 0.57 | Positive Linear |
| Turkey | 1.21 | 0.10 | 0.04 | 0.52 | 1.14 | -0.60 | 0.01 | 0.55 | 0.59 | Positive Linear |
| Laos | 1.10 | 0.02 | -0.01 | 0.59 | 0.95 | -0.43 | 0.01 | 0.55 | 0.56 | Positive Linear |
| Morocco | 1.36 | 0.02 | 0.05 | 1.08 | 0.74 | 0.56 | 0.01 | 0.54 | 0.53 | Positive Linear |
| Algeria | 1.32 | 0.01 | 0.05 | 1.10 | 0.62 | 0.80 | 0.01 | 0.54 | 0.54 | Positive Linear |
| Bosnia and Herzegovina | 1.48 | 0.12 | 0.11 | 0.85 | 0.90 | -0.07 | 0.01 | 0.53 | 0.51 | Positive Linear |
| Vietnam | 1.15 | 0.01 | -0.01 | 0.58 | 0.86 | -0.34 | 0.01 | 0.53 | 0.53 | Positive Linear |
| Cambodia | 1.16 | 0.02 | -0.01 | 0.58 | 0.86 | -0.33 | 0.01 | 0.53 | 0.53 | Positive Linear |
| Macau | 1.17 | 0.02 | 0.00 | 0.58 | 0.82 | -0.29 | 0.01 | 0.53 | 0.52 | Positive Linear |
| Austria | 1.53 | 0.09 | 0.14 | 0.92 | 0.54 | 0.52 | 0.01 | 0.53 | 0.52 | Positive Linear |
| Serbia | 1.48 | 0.11 | 0.11 | 0.85 | 0.86 | -0.01 | 0.01 | 0.52 | 0.51 | Positive Linear |
| Bhutan | 1.09 | 0.01 | 0.00 | 0.63 | 0.90 | -0.33 | 0.01 | 0.52 | 0.52 | Positive Linear |
| Bangladesh | 1.11 | 0.01 | 0.00 | 0.63 | 0.91 | -0.34 | 0.01 | 0.52 | 0.52 | Positive Linear |
| South Korea | 1.21 | 0.01 | 0.00 | 0.57 | 0.70 | -0.16 | 0.01 | 0.51 | 0.50 | Positive Linear |
| Christmas Island | 1.07 | -0.03 | 0.04 | 1.13 | -0.17 | 2.87 | 0.01 | 0.51 | 0.57 | Positive Linear |
| AfghanistanCentral African Republic | 1.23 | 0.01 | 0.00 | 0.57 | 0.72 | -0.18 | 0.01 | 0.50 | 0.49 | Positive Linear |
| Slovenia | 1.53 | 0.11 | 0.16 | 0.89 | 0.45 | 0.59 | 0.01 | 0.50 | 0.49 | Positive Linear |
| AfghanistanCape Verde | 1.01 | 0.01 | 0.02 | 1.01 | 0.88 | 0.29 | 0.03 | 0.50 | 0.48 | Positive Linear |
| Jan Mayen | 1.43 | 0.19 | 0.06 | 0.89 | 1.63 | -0.87 | 0.01 | 0.49 | 0.49 | Positive Linear |
| North Korea | 1.25 | 0.01 | 0.00 | 0.55 | 0.61 | -0.07 | 0.02 | 0.49 | 0.47 | Positive Linear |
| India | 0.93 | -0.01 | 0.04 | 0.88 | -0.21 | 2.39 | 0.01 | 0.48 | 0.54 | Positive Linear |
| AfghanistanCosta Rica | 1.44 | 0.13 | 0.14 | 0.82 | 0.74 | 0.11 | 0.01 | 0.48 | 0.46 | Positive Linear |
| Nepal | 0.95 | -0.01 | 0.04 | 0.86 | -0.21 | 2.39 | 0.02 | 0.48 | 0.54 | Positive Linear |
| Singapore | 1.16 | 0.01 | -0.04 | 0.75 | 1.31 | -0.68 | 0.01 | 0.48 | 0.51 | Positive Linear |
| Greenland | 1.41 | 0.22 | 0.06 | 0.85 | 1.73 | -1.02 | 0.01 | 0.47 | 0.48 | Positive Linear |
| Ivory Coast | 0.58 | -0.02 | 0.01 | 0.65 | -0.32 | 4.58 | 0.01 | 0.46 | 0.67 | Convex |
| Liechtenstein | 1.48 | 0.15 | 0.20 | 0.84 | 0.45 | 0.53 | 0.01 | 0.46 | 0.45 | Positive Linear |
| Switzerland | 1.48 | 0.15 | 0.20 | 0.84 | 0.45 | 0.53 | 0.01 | 0.46 | 0.45 | Positive Linear |
| Monaco | 1.47 | 0.14 | 0.20 | 0.83 | 0.37 | 0.63 | 0.01 | 0.46 | 0.45 | Positive Linear |
| San Marino | 1.47 | 0.13 | 0.16 | 0.80 | 0.59 | 0.30 | 0.01 | 0.46 | 0.44 | Positive Linear |
| Croatia | 1.51 | 0.11 | 0.14 | 0.82 | 0.62 | 0.28 | 0.01 | 0.45 | 0.44 | Positive Linear |
| Brunei | 1.22 | 0.02 | -0.03 | 0.67 | 1.21 | -0.66 | 0.01 | 0.45 | 0.48 | Positive Linear |
| Malaysia | 1.22 | 0.02 | -0.03 | 0.67 | 1.21 | -0.66 | 0.01 | 0.45 | 0.48 | Positive Linear |
| Greece | 1.42 | 0.15 | 0.08 | 0.56 | 1.09 | -0.53 | 0.01 | 0.45 | 0.47 | Positive Linear |
| Libya | 1.23 | 0.11 | 0.06 | 0.51 | 0.89 | -0.37 | 0.01 | 0.45 | 0.45 | Positive Linear |
| Tunisia | 1.46 | 0.09 | 0.12 | 0.90 | 0.64 | 0.38 | 0.01 | 0.44 | 0.43 | Positive Linear |
| Germany | 1.52 | 0.15 | 0.24 | 0.83 | 0.18 | 0.85 | 0.01 | 0.44 | 0.44 | Positive Linear |
| Denmark | 1.54 | 0.22 | 0.30 | 0.78 | 0.30 | 0.60 | 0.02 | 0.44 | 0.43 | Positive Linear |
| Japan | 1.25 | 0.00 | 0.01 | 0.53 | 0.46 | 0.08 | 0.02 | 0.43 | 0.41 | Positive Linear |
| Gibraltar | 1.49 | 0.12 | 0.11 | 0.85 | 0.98 | -0.18 | 0.01 | 0.43 | 0.41 | Positive Linear |
| Bulgaria | 1.50 | 0.14 | 0.10 | 0.65 | 0.96 | -0.36 | 0.01 | 0.43 | 0.42 | Positive Linear |
| Italy | 1.46 | 0.15 | 0.19 | 0.75 | 0.49 | 0.37 | 0.01 | 0.42 | 0.41 | Positive Linear |
| Gambia, The | 0.77 | -0.03 | -0.03 | 0.94 | 1.00 | -0.18 | 0.01 | 0.42 | 0.40 | Positive Linear |
| Guinea-Bissau | 0.77 | -0.03 | -0.03 | 0.94 | 1.00 | -0.18 | 0.01 | 0.42 | 0.40 | Positive Linear |
| Philippines | 1.27 | 0.02 | -0.02 | 0.65 | 1.02 | -0.47 | 0.01 | 0.42 | 0.42 | Positive Linear |
| Indonesia | 1.28 | 0.01 | -0.02 | 0.65 | 1.04 | -0.47 | 0.01 | 0.42 | 0.42 | Positive Linear |
| Cape Verde | 0.81 | -0.02 | -0.02 | 0.92 | 1.09 | -0.56 | 0.01 | 0.41 | 0.39 | Positive Linear |
| Albania | 1.45 | 0.12 | 0.17 | 0.77 | 0.36 | 0.57 | 0.01 | 0.41 | 0.40 | Positive Linear |
| Senegal | 0.77 | -0.03 | -0.03 | 0.94 | 0.93 | 0.03 | 0.01 | 0.41 | 0.39 | Positive Linear |
| Malta | 1.45 | 0.12 | 0.16 | 0.78 | 0.46 | 0.45 | 0.01 | 0.41 | 0.39 | Positive Linear |
| Macedonia | 1.45 | 0.13 | 0.16 | 0.77 | 0.45 | 0.44 | 0.01 | 0.41 | 0.39 | Positive Linear |
| Spain | 1.51 | 0.14 | 0.13 | 0.81 | 0.90 | -0.12 | 0.03 | 0.41 | 0.38 | Positive Linear |
| Luxembourg | 1.52 | 0.18 | 0.24 | 0.78 | 0.36 | 0.56 | 0.01 | 0.40 | 0.39 | Positive Linear |
| Portugal | 1.50 | 0.14 | 0.13 | 0.82 | 0.90 | -0.11 | 0.02 | 0.40 | 0.38 | Positive Linear |
| Netherlands | 1.52 | 0.21 | 0.32 | 0.74 | 0.03 | 0.90 | 0.02 | 0.39 | 0.40 | Positive Linear |
| Iceland | 1.51 | 0.23 | 0.27 | 0.80 | 0.50 | 0.37 | 0.01 | 0.39 | 0.37 | Positive Linear |
| Faroe Islands | 1.51 | 0.23 | 0.27 | 0.80 | 0.50 | 0.37 | 0.02 | 0.39 | 0.37 | Positive Linear |
| Belgium | 1.51 | 0.21 | 0.31 | 0.74 | 0.06 | 0.88 | 0.02 | 0.36 | 0.36 | Positive Linear |
| France | 1.45 | 0.20 | 0.30 | 0.74 | 0.06 | 0.87 | 0.01 | 0.35 | 0.35 | Positive Linear |
| Pacific Islands (Palau) | 1.28 | 0.01 | -0.01 | 0.60 | 0.74 | -0.16 | 0.01 | 0.34 | 0.32 | Positive Linear |
| Andorra | 1.50 | 0.19 | 0.21 | 0.72 | 0.58 | 0.19 | 0.03 | 0.34 | 0.32 | Positive Linear |
| Guernsey | 1.45 | 0.21 | 0.32 | 0.74 | 0.04 | 0.88 | 0.01 | 0.33 | 0.33 | Positive Linear |
| Jersey | 1.45 | 0.21 | 0.32 | 0.74 | 0.04 | 0.88 | 0.01 | 0.33 | 0.33 | Positive Linear |
| Guam | 1.24 | -0.01 | -0.01 | 0.60 | 0.58 | 0.02 | 0.01 | 0.31 | 0.28 | Positive Linear |
| Burkina Faso | 0.65 | -0.02 | 0.00 | 0.79 | 0.19 | 2.85 | 0.02 | 0.31 | 0.32 | Positive Linear |
| Niger | 0.65 | -0.02 | 0.00 | 0.78 | 0.16 | 2.98 | 0.01 | 0.30 | 0.32 | Positive Linear |
| Seychelles | 0.61 | 0.00 | 0.00 | 0.44 | 0.31 | 0.57 | 0.03 | 0.29 | 0.27 | Positive Linear |
| Northern Mariana Islands | 1.25 | -0.01 | 0.02 | 0.48 | 0.22 | 0.31 | 0.02 | 0.27 | 0.27 | Positive Linear |
| Man, Isle of | 1.45 | 0.28 | 0.42 | 0.67 | -0.19 | 1.03 | 0.01 | 0.27 | 0.28 | Positive Linear |
| United Kingdom | 1.45 | 0.28 | 0.42 | 0.67 | -0.19 | 1.03 | 0.02 | 0.27 | 0.28 | Positive Linear |
| Ireland | 1.44 | 0.27 | 0.42 | 0.69 | -0.18 | 1.03 | 0.02 | 0.26 | 0.27 | Positive Linear |
| Kenya | 0.47 | 0.00 | 0.01 | 0.26 | -0.19 | 2.95 | 0.02 | 0.25 | 0.37 | Convex |
| Federated States of Micronesia | 1.15 | -0.03 | 0.03 | 0.49 | 0.08 | 0.46 | 0.04 | 0.25 | 0.26 | Positive Linear |
| Somalia | 0.63 | 0.01 | 0.01 | 0.41 | 0.29 | 0.51 | 0.01 | 0.24 | 0.22 | Positive Linear |
| Tajikistan | 0.89 | 0.04 | 0.02 | 0.30 | 0.82 | -1.25 | 0.02 | 0.24 | 0.30 | Positive Linear |
| Australia | 1.13 | -0.07 | 0.14 | 1.01 | -1.72 | 6.46 | 0.01 | 0.24 | 0.50 | Convex |
| Marshall Islands | 1.13 | -0.04 | -0.03 | 0.57 | 0.52 | 0.06 | 0.02 | 0.24 | 0.21 | Positive Linear |
| Kyrgyzstan | 0.90 | 0.04 | 0.02 | 0.30 | 0.84 | -1.29 | 0.02 | 0.23 | 0.30 | Positive Linear |
| Baker Island | 1.16 | -0.04 | -0.02 | 0.55 | 0.44 | 0.12 | 0.02 | 0.23 | 0.20 | Positive Linear |
| Howland Island | 1.16 | -0.04 | -0.02 | 0.55 | 0.44 | 0.12 | 0.01 | 0.23 | 0.20 | Positive Linear |
| Mauritius | 0.56 | 0.00 | 0.00 | 0.34 | 0.36 | -0.10 | 0.03 | 0.21 | 0.18 | Positive Linear |
| Jarvis Island | 1.12 | -0.06 | -0.01 | 0.68 | 0.25 | 0.65 | 0.02 | 0.19 | 0.18 | Positive Linear |
| Kiribati | 1.05 | -0.04 | 0.04 | 0.49 | -0.10 | 0.90 | 0.04 | 0.19 | 0.22 | Positive Linear |
| Cameroon | 0.44 | 0.00 | 0.00 | 0.12 | 0.09 | 0.20 | 0.03 | 0.19 | 0.16 | Positive Linear |
| Papua New Guinea | 1.18 | -0.10 | 0.02 | 0.71 | 0.34 | 0.64 | 0.02 | 0.17 | 0.15 | Positive Linear |
| French Polynesia | 0.92 | -0.03 | 0.04 | 0.46 | -0.34 | 1.68 | 0.02 | 0.15 | 0.24 | Positive Linear |
| Peru | 0.58 | 0.03 | 0.03 | 0.31 | 0.26 | 0.19 | 0.02 | 0.13 | 0.10 | Positive Linear |
| Nigeria | 0.47 | 0.00 | 0.00 | 0.28 | 0.06 | 1.61 | 0.02 | 0.12 | 0.10 | Positive Linear |
| Sao Tome and Principe | 0.39 | 0.00 | 0.00 | 0.05 | 0.07 | -0.20 | 0.03 | 0.09 | 0.06 | Positive Linear |
| Barbados | 0.59 | 0.04 | 0.05 | 0.20 | -0.20 | 1.50 | 0.02 | 0.02 | 0.00 | Undefined Complex |
| New Zealand | 1.02 | 0.04 | 0.23 | 0.34 | -2.46 | 7.26 | 0.02 | 0.00 | 0.20 | Convex |
| El Salvador | 0.48 | 0.03 | 0.00 | 0.25 | 1.95 | -15.25 | 0.01 | -0.01 | 0.02 | Undefined Complex |
| Costa Rica | 0.50 | 0.03 | 0.00 | 0.25 | 1.85 | -14.42 | 0.03 | -0.01 | 0.01 | Undefined Complex |
| Belize | 0.49 | 0.03 | 0.00 | 0.24 | 1.98 | -15.61 | 0.01 | -0.02 | 0.02 | Undefined Complex |
| Guatemala | 0.49 | 0.03 | 0.00 | 0.24 | 1.98 | -15.61 | 0.01 | -0.02 | 0.02 | Undefined Complex |
| Cayman Islands | 0.49 | 0.03 | 0.00 | 0.24 | 1.98 | -15.61 | 0.02 | -0.02 | 0.02 | Undefined Complex |
| Panama | 0.48 | 0.03 | 0.00 | 0.27 | 2.06 | -17.21 | 0.02 | -0.02 | 0.02 | Undefined Complex |
| Honduras | 0.49 | 0.03 | 0.00 | 0.23 | 1.94 | -15.36 | 0.01 | -0.02 | 0.02 | Undefined Complex |
| Nicaragua | 0.49 | 0.03 | 0.00 | 0.23 | 1.94 | -15.36 | 0.02 | -0.02 | 0.02 | Undefined Complex |
| Haiti | 0.48 | 0.03 | 0.00 | 0.32 | 2.17 | -19.15 | 0.02 | -0.02 | 0.03 | Undefined Complex |
| Jamaica | 0.48 | 0.03 | 0.00 | 0.32 | 2.17 | -19.15 | 0.02 | -0.02 | 0.03 | Undefined Complex |
| Cuba | 0.48 | 0.03 | 0.00 | 0.32 | 2.17 | -19.15 | 0.04 | -0.02 | 0.03 | Undefined Complex |
| **Expenditure on R&D (% of GDP) and Total Patent application** | | | | | | | | | | |
| Chad | 0.48 | 0.00 | 0.00 | 0.01 | 0.00 | 0.00 | 0.01 | 0.88 | 0.89 | Positive Linear |
| French Guiana | 0.27 | 0.00 | 0.00 | 0.07 | 0.01 | 0.19 | 0.01 | 0.87 | 0.91 | Convex |
| Ethiopia | 0.52 | 0.00 | 0.00 | 0.01 | 0.00 | 0.00 | 0.01 | 0.84 | 0.84 | Positive Linear |
| Sudan | 0.56 | 0.00 | 0.00 | 0.01 | 0.00 | 0.00 | 0.01 | 0.83 | 0.83 | Positive Linear |
| Suriname | 0.28 | 0.00 | 0.00 | 0.06 | 0.00 | 0.23 | 0.01 | 0.83 | 0.90 | Convex |
| Brazil | 0.30 | 0.00 | 0.00 | 0.06 | 0.01 | 0.21 | 0.01 | 0.83 | 0.91 | Convex |
| Barbados | 0.27 | 0.00 | 0.00 | 0.06 | -0.01 | 0.27 | 0.01 | 0.82 | 0.94 | Convex |
| Trinidad and Tobago | 0.27 | 0.00 | 0.00 | 0.06 | -0.01 | 0.27 | 0.01 | 0.82 | 0.94 | Convex |
| Guyana | 0.28 | 0.00 | 0.00 | 0.06 | -0.01 | 0.27 | 0.01 | 0.79 | 0.91 | Convex |
| Mexico | 0.43 | -0.02 | 0.01 | 0.59 | -0.52 | 1.96 | 0.01 | 0.77 | 0.97 | Convex |
| Peru | 0.31 | 0.00 | 0.00 | 0.06 | -0.01 | 0.25 | 0.01 | 0.77 | 0.89 | Convex |
| Bolivia | 0.31 | 0.00 | 0.00 | 0.06 | -0.01 | 0.26 | 0.01 | 0.75 | 0.88 | Convex |
| Paraguay | 0.31 | 0.00 | 0.00 | 0.06 | -0.01 | 0.26 | 0.01 | 0.75 | 0.88 | Convex |
| Kiribati | 0.77 | -0.05 | 0.00 | 0.46 | 0.03 | 0.66 | 0.01 | 0.59 | 0.67 | Convex |
| Egypt | 0.64 | 0.00 | 0.00 | 0.01 | 0.00 | 0.00 | 0.01 | 0.57 | 0.55 | Positive Linear |
| Fiji | 0.73 | -0.02 | 0.01 | 0.25 | 0.06 | 0.33 | 0.01 | 0.56 | 0.63 | Convex |
| American Samoa | 0.73 | -0.02 | 0.01 | 0.25 | 0.06 | 0.33 | 0.01 | 0.56 | 0.63 | Convex |
| Vanuatu | 0.73 | -0.02 | 0.01 | 0.25 | 0.06 | 0.33 | 0.01 | 0.56 | 0.63 | Convex |
| Tonga | 0.73 | -0.02 | 0.01 | 0.25 | 0.06 | 0.33 | 0.01 | 0.56 | 0.63 | Convex |
| Western Samoa | 0.73 | -0.02 | 0.01 | 0.25 | 0.06 | 0.33 | 0.01 | 0.56 | 0.63 | Convex |
| French Polynesia | 0.71 | -0.05 | 0.01 | 0.45 | -0.31 | 1.61 | 0.01 | 0.55 | 0.90 | Convex |
| Jarvis Island | 0.81 | -0.06 | 0.01 | 0.44 | -0.04 | 0.71 | 0.01 | 0.53 | 0.62 | Convex |
| Cyprus | 0.67 | 0.00 | 0.00 | 0.01 | 0.00 | 0.00 | 0.01 | 0.53 | 0.52 | Positive Linear |
| Libya | 0.70 | 0.00 | 0.00 | 0.01 | 0.01 | 0.00 | 0.03 | 0.48 | 0.46 | Positive Linear |
| Cape Verde | 0.53 | 0.00 | 0.00 | 0.01 | 0.00 | 0.03 | 0.01 | 0.43 | 0.48 | Positive Linear |
| Heard Island & McDonald Islands | 0.49 | 0.00 | 0.00 | 0.02 | 0.02 | 0.00 | 0.01 | 0.40 | 0.38 | Positive Linear |
| Yemen | 0.60 | 0.00 | 0.00 | 0.00 | 0.00 | 0.00 | 0.01 | 0.39 | 0.37 | Positive Linear |
| Djibouti | 0.59 | 0.00 | 0.00 | 0.00 | 0.00 | 0.00 | 0.01 | 0.38 | 0.36 | Positive Linear |
| Solomon Islands | 0.76 | -0.01 | 0.01 | 0.24 | 0.06 | 0.30 | 0.01 | 0.38 | 0.41 | Positive Linear |
| Nauru | 0.77 | -0.01 | 0.01 | 0.25 | 0.05 | 0.31 | 0.01 | 0.38 | 0.41 | Positive Linear |
| Tuvalu | 0.77 | -0.01 | 0.01 | 0.25 | 0.05 | 0.31 | 0.01 | 0.38 | 0.41 | Positive Linear |
| Eritrea | 0.61 | 0.00 | 0.00 | 0.00 | 0.00 | 0.00 | 0.01 | 0.37 | 0.36 | Positive Linear |
| Iran | 0.63 | 0.00 | 0.00 | 0.00 | 0.01 | 0.00 | 0.01 | 0.35 | 0.33 | Positive Linear |
| Bahrain | 0.62 | 0.00 | 0.00 | 0.00 | 0.01 | 0.00 | 0.01 | 0.35 | 0.32 | Positive Linear |
| Qatar | 0.62 | 0.00 | 0.00 | 0.00 | 0.01 | 0.00 | 0.01 | 0.35 | 0.32 | Positive Linear |
| United Arab Emirates | 0.62 | 0.00 | 0.00 | 0.00 | 0.01 | 0.00 | 0.01 | 0.35 | 0.32 | Positive Linear |
| Saudi Arabia | 0.62 | 0.00 | 0.00 | 0.00 | 0.01 | 0.00 | 0.01 | 0.34 | 0.32 | Positive Linear |
| Kuwait | 0.63 | 0.00 | 0.00 | 0.00 | 0.00 | 0.00 | 0.01 | 0.34 | 0.31 | Positive Linear |
| French Southern & Antarctic Lands | 0.47 | 0.00 | 0.00 | 0.02 | 0.02 | 0.01 | 0.01 | 0.33 | 0.31 | Positive Linear |
| United States | 0.54 | -0.01 | 0.02 | 0.27 | -0.83 | 2.09 | 0.01 | 0.32 | 0.58 | Convex |
| Cook Islands | 0.64 | 0.00 | -0.01 | 0.17 | 0.31 | -0.37 | 0.01 | 0.31 | 0.31 | Positive Linear |
| Johnston Atoll | 0.91 | 0.00 | 0.00 | 0.26 | 0.25 | 0.01 | 0.01 | 0.31 | 0.28 | Positive Linear |
| Iraq | 0.66 | 0.00 | 0.00 | 0.00 | 0.00 | 0.00 | 0.01 | 0.31 | 0.28 | Positive Linear |
| West Bank | 0.66 | 0.00 | 0.00 | 0.00 | 0.00 | 0.00 | 0.01 | 0.30 | 0.28 | Positive Linear |
| Gaza Strip | 0.66 | 0.00 | 0.00 | 0.00 | 0.00 | 0.00 | 0.01 | 0.30 | 0.28 | Positive Linear |
| Israel | 0.66 | 0.00 | 0.00 | 0.00 | 0.00 | 0.00 | 0.01 | 0.30 | 0.28 | Positive Linear |
| Jordan | 0.68 | 0.00 | 0.00 | 0.00 | 0.00 | 0.00 | 0.01 | 0.30 | 0.28 | Positive Linear |
| Azerbaijan | 0.67 | 0.00 | 0.00 | 0.00 | 0.00 | 0.00 | 0.01 | 0.30 | 0.28 | Positive Linear |
| Papua New Guinea | 0.74 | -0.01 | 0.02 | 0.19 | -0.03 | 0.37 | 0.01 | 0.30 | 0.36 | Positive Linear |
| New Caledonia | 0.65 | 0.00 | -0.01 | 0.17 | 0.28 | -0.31 | 0.01 | 0.29 | 0.29 | Positive Linear |
| Colombia | 0.65 | 0.00 | -0.01 | 0.17 | 0.28 | -0.31 | 0.01 | 0.29 | 0.29 | Positive Linear |
| Armenia | 0.67 | 0.00 | 0.00 | 0.00 | 0.00 | 0.00 | 0.01 | 0.29 | 0.27 | Positive Linear |
| Lebanon | 0.68 | 0.00 | 0.00 | 0.00 | 0.00 | 0.00 | 0.01 | 0.28 | 0.27 | Positive Linear |
| Georgia | 0.67 | 0.00 | 0.00 | 0.00 | 0.00 | 0.01 | 0.01 | 0.28 | 0.29 | Positive Linear |
| Midway Islands | 0.93 | 0.01 | 0.01 | 0.25 | 0.26 | -0.01 | 0.01 | 0.28 | 0.26 | Positive Linear |
| Syria | 0.68 | 0.00 | 0.00 | 0.00 | 0.00 | 0.00 | 0.02 | 0.28 | 0.27 | Positive Linear |
| New Zealand | 0.64 | 0.00 | -0.01 | 0.16 | 0.32 | -0.42 | 0.01 | 0.28 | 0.29 | Positive Linear |
| Turkey | 0.69 | 0.00 | 0.00 | 0.00 | 0.00 | 0.01 | 0.01 | 0.27 | 0.29 | Positive Linear |
| Baker Island | 0.87 | 0.02 | 0.04 | 0.15 | 0.01 | 0.16 | 0.01 | 0.27 | 0.29 | Positive Linear |
| Howland Island | 0.87 | 0.02 | 0.04 | 0.15 | 0.01 | 0.16 | 0.01 | 0.27 | 0.29 | Positive Linear |
| Belize | 0.21 | 0.00 | 0.00 | 0.04 | -0.05 | 0.85 | 0.01 | 0.27 | 0.35 | Positive Linear |
| Guatemala | 0.21 | 0.00 | 0.00 | 0.04 | -0.05 | 0.85 | 0.01 | 0.27 | 0.35 | Positive Linear |
| Cayman Islands | 0.21 | 0.00 | 0.00 | 0.04 | -0.05 | 0.85 | 0.01 | 0.27 | 0.35 | Positive Linear |
| Honduras | 0.22 | 0.00 | 0.00 | 0.04 | -0.05 | 0.85 | 0.01 | 0.27 | 0.35 | Positive Linear |
| Nicaragua | 0.22 | 0.00 | 0.00 | 0.04 | -0.05 | 0.85 | 0.01 | 0.27 | 0.35 | Positive Linear |
| Costa Rica | 0.22 | 0.00 | 0.00 | 0.04 | -0.05 | 0.84 | 0.01 | 0.26 | 0.34 | Positive Linear |
| El Salvador | 0.22 | 0.00 | 0.00 | 0.04 | -0.05 | 0.84 | 0.01 | 0.26 | 0.34 | Positive Linear |
| Antigua and Barbuda | 0.20 | 0.00 | 0.00 | 0.02 | 0.05 | -0.36 | 0.01 | 0.24 | 0.30 | Positive Linear |
| Anguilla | 0.20 | 0.00 | 0.00 | 0.02 | 0.05 | -0.36 | 0.01 | 0.24 | 0.30 | Positive Linear |
| Guadeloupe | 0.20 | 0.00 | 0.00 | 0.02 | 0.05 | -0.36 | 0.01 | 0.24 | 0.30 | Positive Linear |
| Montserrat | 0.20 | 0.00 | 0.00 | 0.02 | 0.05 | -0.36 | 0.01 | 0.24 | 0.30 | Positive Linear |
| St. Kitts and Nevis | 0.20 | 0.00 | 0.00 | 0.02 | 0.05 | -0.36 | 0.01 | 0.24 | 0.30 | Positive Linear |
| Puerto Rico | 0.20 | 0.00 | 0.00 | 0.02 | 0.05 | -0.36 | 0.01 | 0.24 | 0.30 | Positive Linear |
| Federated States of Micronesia | 0.89 | 0.02 | 0.04 | 0.15 | 0.05 | 0.11 | 0.01 | 0.23 | 0.36 | Positive Linear |
| Falkland Islands (Islas Malvinas) | 0.44 | 0.00 | 0.00 | 0.02 | 0.07 | -0.12 | 0.01 | 0.20 | 0.42 | Concave |
| Chile | 0.42 | 0.00 | 0.00 | 0.02 | 0.08 | -0.14 | 0.01 | 0.20 | 0.45 | Concave |
| Argentina | 0.39 | 0.00 | 0.00 | 0.02 | 0.08 | -0.15 | 0.01 | 0.20 | 0.46 | Concave |
| Uruguay | 0.39 | 0.00 | 0.00 | 0.02 | 0.08 | -0.15 | 0.01 | 0.20 | 0.46 | Concave |
| Marshall Islands | 0.90 | 0.02 | 0.05 | 0.14 | -0.04 | 0.18 | 0.01 | 0.18 | 0.20 | Positive Linear |
| Guam | 0.92 | 0.02 | 0.04 | 0.12 | -0.03 | 0.18 | 0.01 | 0.16 | 0.19 | Positive Linear |
| Morocco | 0.71 | 0.00 | 0.00 | 0.01 | 0.01 | -0.01 | 0.01 | 0.16 | 0.16 | Positive Linear |
| Dominican Republic | 0.20 | 0.00 | 0.00 | 0.01 | 0.03 | -0.16 | 0.01 | 0.15 | 0.17 | Positive Linear |
| Christmas Island | 0.94 | -0.02 | 0.06 | 0.59 | -0.68 | 2.81 | 0.01 | 0.15 | 0.22 | Positive Linear |
| Dominica | 0.20 | 0.00 | 0.00 | 0.01 | 0.07 | -0.54 | 0.01 | 0.15 | 0.32 | Concave |
| Grenada | 0.20 | 0.00 | 0.00 | 0.01 | 0.07 | -0.54 | 0.01 | 0.15 | 0.32 | Concave |
| Martinique | 0.20 | 0.00 | 0.00 | 0.01 | 0.07 | -0.54 | 0.01 | 0.15 | 0.32 | Concave |
| St. Lucia | 0.20 | 0.00 | 0.00 | 0.01 | 0.07 | -0.54 | 0.01 | 0.15 | 0.32 | Concave |
| St. Vincent and the Grenadines | 0.20 | 0.00 | 0.00 | 0.01 | 0.07 | -0.54 | 0.01 | 0.15 | 0.32 | Concave |
| Venezuela | 0.20 | 0.00 | 0.00 | 0.01 | 0.07 | -0.54 | 0.01 | 0.13 | 0.31 | Concave |
| Aruba | 0.21 | 0.00 | 0.00 | 0.01 | 0.05 | -0.33 | 0.01 | 0.12 | 0.22 | Positive Linear |
| Bermuda | 0.19 | 0.00 | 0.00 | 0.01 | 0.02 | -0.09 | 0.01 | 0.12 | 0.10 | Positive Linear |
| Colombia | 0.21 | 0.00 | 0.00 | 0.01 | 0.05 | -0.35 | 0.01 | 0.11 | 0.22 | Positive Linear |
| Macau | 1.11 | 0.03 | 0.00 | 0.30 | 0.72 | -0.50 | 0.01 | 0.11 | 0.11 | Positive Linear |
| South Korea | 1.14 | 0.03 | 0.00 | 0.29 | 0.64 | -0.42 | 0.01 | 0.10 | 0.09 | Positive Linear |
| Central African Republic | 1.15 | 0.03 | 0.00 | 0.29 | 0.62 | -0.40 | 0.02 | 0.09 | 0.09 | Positive Linear |
| North Korea | 1.18 | 0.03 | 0.01 | 0.29 | 0.60 | -0.38 | 0.02 | 0.09 | 0.08 | Positive Linear |
| Brunei | 1.15 | 0.03 | 0.01 | 0.28 | 0.58 | -0.36 | 0.01 | 0.08 | 0.07 | Positive Linear |
| Malaysia | 1.15 | 0.03 | 0.01 | 0.28 | 0.57 | -0.36 | 0.02 | 0.08 | 0.07 | Positive Linear |
| Northern Mariana Islands | 1.02 | 0.02 | 0.00 | 0.29 | 0.46 | -0.21 | 0.01 | 0.08 | 0.05 | Positive Linear |
| Japan | 1.17 | 0.03 | 0.01 | 0.27 | 0.50 | -0.28 | 0.01 | 0.08 | 0.06 | Positive Linear |
| Philippines | 1.14 | 0.03 | 0.01 | 0.27 | 0.51 | -0.29 | 0.01 | 0.08 | 0.06 | Positive Linear |
| Indonesia | 1.15 | 0.03 | 0.01 | 0.27 | 0.50 | -0.29 | 0.01 | 0.08 | 0.05 | Positive Linear |
| Pacific Islands (Palau) | 1.07 | 0.02 | 0.01 | 0.26 | 0.39 | -0.16 | 0.01 | 0.07 | 0.04 | Positive Linear |
| Haiti | 0.19 | 0.00 | 0.00 | 0.00 | 0.01 | -0.07 | 0.01 | 0.06 | 0.06 | Positive Linear |
| Jamaica | 0.19 | 0.00 | 0.00 | 0.00 | 0.01 | -0.07 | 0.01 | 0.06 | 0.06 | Positive Linear |
| Bahamas, The | 0.19 | 0.00 | 0.00 | 0.00 | 0.01 | -0.07 | 0.01 | 0.06 | 0.06 | Positive Linear |
| Cuba | 0.19 | 0.00 | 0.00 | 0.00 | 0.01 | -0.07 | 0.01 | 0.06 | 0.06 | Positive Linear |
| Ecuador | 0.24 | 0.00 | 0.00 | 0.01 | 0.05 | -0.40 | 0.02 | 0.06 | 0.15 | Positive Linear |
| Mauritania | 0.46 | 0.00 | 0.00 | 0.00 | 0.01 | -0.02 | 0.02 | 0.05 | 0.14 | Positive Linear |
| Canada | 0.74 | -0.01 | 0.00 | 0.09 | -0.06 | 0.25 | 0.02 | 0.05 | 0.03 | Positive Linear |
| Namibia | 0.41 | 0.00 | 0.00 | 0.00 | 0.01 | -0.01 | 0.01 | 0.05 | 0.04 | Positive Linear |
| Swaziland | 0.42 | 0.00 | 0.00 | 0.00 | 0.01 | -0.01 | 0.02 | 0.05 | 0.02 | Undefined Complex |
| Australia | 0.68 | 0.01 | -0.01 | 0.07 | 0.34 | -0.62 | 0.01 | 0.03 | 0.11 | Undefined Complex |
| Bouvet Island | 0.45 | 0.00 | 0.00 | 0.00 | 0.01 | -0.03 | 0.01 | 0.02 | 0.08 | Undefined Complex |
| Panama | 0.20 | 0.00 | 0.00 | 0.00 | 0.01 | -0.10 | 0.01 | 0.02 | 0.05 | Undefined Complex |
| Mauritius | 0.35 | 0.00 | 0.00 | 0.01 | 0.00 | 0.05 | 0.02 | 0.02 | 0.00 | Undefined Complex |
| Cape Verde | 0.43 | 0.00 | 0.00 | 0.00 | 0.01 | -0.03 | 0.01 | -0.01 | 0.08 | Undefined Complex |
| **Expenditure on R&D (% of GDP) and Trademark Application** | | | | | | | | | | |
| Antigua and Barbuda | 0.34 | 0.00 | -0.02 | 0.61 | 1.49 | -9.23 | 0.01 | 0.26 | 0.30 | Positive Linear |
| Argentina | 0.66 | 0.02 | -0.05 | 0.47 | 2.24 | -4.17 | 0.01 | 0.18 | 0.46 | Concave |
| Belize | 0.43 | -0.02 | 0.03 | 1.16 | -1.44 | 23.29 | 0.01 | 0.31 | 0.41 | Convex |
| Bolivia | 0.55 | -0.03 | 0.03 | 1.53 | -0.19 | 6.82 | 0.01 | 0.74 | 0.85 | Convex |
| Myanmar (Burma) | 1.09 | 0.06 | 0.08 | 0.30 | 0.10 | 0.23 | 0.01 | 0.15 | 0.13 | Positive Linear |
| Solomon Islands | 0.88 | 0.04 | 0.07 | 0.41 | 0.22 | 0.31 | 0.01 | 0.42 | 0.42 | Positive Linear |
| Brazil | 0.53 | -0.03 | 0.01 | 1.66 | 0.23 | 5.48 | 0.01 | 0.80 | 0.87 | Convex |
| Cambodia | 1.17 | 0.07 | 0.09 | 0.32 | 0.11 | 0.24 | 0.01 | 0.21 | 0.19 | Positive Linear |
| China | 1.15 | 0.06 | 0.07 | 0.34 | 0.24 | 0.13 | 0.01 | 0.25 | 0.22 | Positive Linear |
| Chile | 0.69 | 0.02 | -0.04 | 0.47 | 2.15 | -3.99 | 0.01 | 0.18 | 0.45 | Concave |
| Cape Verde | 0.62 | 0.02 | 0.01 | 0.10 | 0.29 | -0.62 | 0.01 | 0.10 | 0.13 | Positive Linear |
| Dominica | 0.35 | 0.00 | -0.03 | 0.56 | 1.96 | -13.27 | 0.01 | 0.27 | 0.37 | Concave |
| Dominican Republic | 0.34 | 0.00 | 0.00 | 0.38 | 0.75 | -3.72 | 0.01 | 0.17 | 0.16 | Positive Linear |
| Egypt | 0.86 | 0.03 | 0.01 | 0.00 | 0.23 | -0.22 | 0.02 | -0.04 | 0.02 | Undefined Complex |
| Fiji | 0.90 | 0.04 | 0.06 | 0.41 | 0.24 | 0.29 | 0.01 | 0.45 | 0.45 | Positive Linear |
| Gambia, The | 0.63 | 0.01 | 0.01 | 0.11 | 0.20 | -0.31 | 0.02 | 0.22 | 0.22 | Positive Linear |
| Grenada | 0.35 | 0.00 | -0.03 | 0.56 | 1.96 | -13.27 | 0.01 | 0.27 | 0.37 | Concave |
| Guatemala | 0.43 | -0.02 | 0.03 | 1.16 | -1.44 | 23.29 | 0.02 | 0.31 | 0.41 | Convex |
| Guinea | 0.58 | 0.01 | 0.01 | 0.18 | -0.05 | 0.87 | 0.01 | 0.47 | 0.55 | Convex |
| Guyana | 0.51 | -0.03 | 0.02 | 1.66 | -0.12 | 6.66 | 0.01 | 0.79 | 0.89 | Convex |
| Honduras | 0.43 | -0.02 | 0.03 | 1.16 | -1.42 | 23.19 | 0.01 | 0.31 | 0.41 | Convex |
| Indonesia | 1.07 | 0.05 | 0.08 | 0.32 | 0.02 | 0.36 | 0.01 | 0.58 | 0.65 | Convex |
| Kiribati | 1.01 | -0.04 | 0.06 | 0.84 | 0.08 | 1.16 | 0.01 | 0.44 | 0.48 | Positive Linear |
| Laos | 1.14 | 0.07 | 0.08 | 0.33 | 0.14 | 0.22 | 0.01 | 0.22 | 0.20 | Positive Linear |
| Mongolia | 1.15 | 0.06 | 0.06 | 0.38 | 0.39 | -0.01 | 0.01 | 0.27 | 0.24 | Positive Linear |
| Mali | 0.60 | 0.01 | 0.01 | 0.18 | -0.06 | 0.89 | 0.01 | 0.44 | 0.52 | Convex |
| Morocco | 0.90 | 0.01 | 0.01 | 0.11 | 0.17 | -0.09 | 0.01 | 0.22 | 0.20 | Positive Linear |
| Mauritania | 0.64 | 0.01 | 0.01 | 0.11 | 0.21 | -0.31 | 0.02 | 0.22 | 0.22 | Positive Linear |
| Federated States of Micronesia | 0.97 | 0.05 | 0.07 | 0.38 | 0.26 | 0.14 | 0.01 | 0.61 | 0.61 | Positive Linear |
| Andorra | 1.04 | 0.01 | -0.01 | 0.13 | 0.29 | -0.92 | 0.01 | 0.15 | 0.15 | Positive Linear |
| Australia | 0.84 | 0.07 | 0.04 | 0.15 | 0.55 | -0.95 | 0.01 | 0.11 | 0.21 | Positive Linear |
| Barbados | 0.46 | -0.04 | 0.01 | 1.69 | -0.06 | 6.55 | 0.01 | 0.84 | 0.94 | Convex |
| Brunei | 1.17 | 0.07 | 0.09 | 0.30 | 0.08 | 0.27 | 0.01 | 0.20 | 0.19 | Positive Linear |
| Canada | 1.02 | 0.02 | 0.01 | 0.23 | 0.29 | -0.10 | 0.01 | 0.07 | 0.03 | Positive Linear |
| Cyprus | 0.88 | 0.03 | 0.02 | 0.00 | 0.15 | -0.15 | 0.01 | -0.04 | -0.03 | Undefined Complex |
| France | 1.04 | 0.02 | -0.02 | 0.10 | 0.36 | -0.33 | 0.02 | 0.07 | 0.10 | Positive Linear |
| Germany | 1.01 | 0.00 | -0.03 | 0.12 | 0.34 | -0.29 | 0.01 | 0.13 | 0.14 | Positive Linear |
| Italy | 1.01 | 0.00 | -0.01 | 0.13 | 0.19 | -0.09 | 0.02 | 0.22 | 0.20 | Positive Linear |
| Japan | 1.08 | 0.05 | 0.07 | 0.34 | 0.17 | 0.21 | 0.01 | 0.60 | 0.61 | Positive Linear |
| North Korea | 1.18 | 0.07 | 0.08 | 0.33 | 0.25 | 0.10 | 0.01 | 0.23 | 0.20 | Positive Linear |
| South Korea | 1.14 | 0.07 | 0.08 | 0.32 | 0.20 | 0.15 | 0.01 | 0.22 | 0.20 | Positive Linear |
| Liechtenstein | 1.05 | 0.01 | -0.01 | 0.11 | 0.33 | -0.30 | 0.01 | 0.10 | 0.13 | Positive Linear |
| Luxembourg | 1.04 | 0.01 | -0.02 | 0.11 | 0.33 | -0.29 | 0.01 | 0.10 | 0.12 | Positive Linear |
| Monaco | 1.04 | 0.01 | -0.01 | 0.10 | 0.32 | -0.30 | 0.02 | 0.10 | 0.12 | Positive Linear |
| Aruba | 0.36 | 0.00 | -0.01 | 0.46 | 1.18 | -6.96 | 0.01 | 0.24 | 0.27 | Positive Linear |
| American Samoa | 0.90 | 0.04 | 0.06 | 0.41 | 0.24 | 0.29 | 0.01 | 0.45 | 0.45 | Positive Linear |
| Cayman Islands | 0.43 | -0.02 | 0.03 | 1.16 | -1.44 | 23.29 | 0.01 | 0.31 | 0.41 | Convex |
| Northern Mariana Islands | 0.99 | 0.05 | 0.06 | 0.37 | 0.26 | 0.14 | 0.01 | 0.68 | 0.69 | Positive Linear |
| French Polynesia | 0.96 | -0.05 | 0.07 | 0.97 | -0.60 | 3.31 | 0.01 | 0.50 | 0.79 | Convex |
| Gibraltar | 1.05 | 0.01 | -0.01 | 0.13 | 0.33 | -0.28 | 0.02 | 0.17 | 0.18 | Positive Linear |
| Guam | 0.99 | 0.05 | 0.07 | 0.36 | 0.18 | 0.21 | 0.01 | 0.66 | 0.68 | Positive Linear |
| Macau | 1.14 | 0.07 | 0.08 | 0.33 | 0.16 | 0.20 | 0.01 | 0.22 | 0.20 | Positive Linear |
| Anguilla | 0.34 | 0.00 | -0.02 | 0.61 | 1.49 | -9.23 | 0.01 | 0.26 | 0.30 | Positive Linear |
| Cook Islands | 0.84 | 0.06 | 0.05 | 0.32 | 0.55 | -0.60 | 0.01 | 0.16 | 0.14 | Positive Linear |
| Jarvis Island | 1.04 | -0.03 | 0.08 | 0.80 | -0.07 | 1.30 | 0.01 | 0.39 | 0.45 | Positive Linear |
| French Guiana | 0.47 | -0.03 | 0.00 | 1.80 | 0.66 | 4.10 | 0.01 | 0.87 | 0.89 | Convex |
| Falkland Islands (Islas Malvinas) | 0.73 | 0.02 | -0.03 | 0.45 | 1.77 | -3.20 | 0.01 | 0.17 | 0.36 | Concave |
| Baker Island | 0.97 | 0.05 | 0.08 | 0.38 | 0.20 | 0.19 | 0.01 | 0.56 | 0.56 | Positive Linear |
| French Southern & Antarctic Lands | 0.67 | 0.01 | 0.00 | 0.19 | 0.30 | -0.25 | 0.02 | 0.24 | 0.22 | Positive Linear |
| Guadeloupe | 0.34 | 0.00 | -0.02 | 0.61 | 1.49 | -9.23 | 0.01 | 0.26 | 0.30 | Positive Linear |
| Heard Island & McDonald Islands | 0.69 | 0.01 | 0.00 | 0.15 | 0.33 | -0.39 | 0.01 | 0.21 | 0.21 | Positive Linear |
| Howland Island | 0.97 | 0.05 | 0.08 | 0.38 | 0.20 | 0.19 | 0.01 | 0.56 | 0.56 | Positive Linear |
| Johnston Atoll | 1.10 | 0.03 | 0.03 | 0.57 | 0.61 | -0.03 | 0.01 | 0.36 | 0.33 | Positive Linear |
| Christmas Island | 0.99 | 0.09 | 0.04 | 0.11 | 0.82 | -1.58 | 0.01 | -0.02 | 0.01 | Undefined Complex |
| Martinique | 0.35 | 0.00 | -0.03 | 0.56 | 1.96 | -13.27 | 0.01 | 0.27 | 0.37 | Concave |
| Montserrat | 0.34 | 0.00 | -0.02 | 0.61 | 1.49 | -9.23 | 0.01 | 0.26 | 0.30 | Positive Linear |
| Midway Islands | 1.04 | 0.02 | 0.00 | 0.60 | 0.71 | -0.13 | 0.01 | 0.41 | 0.39 | Positive Linear |
| Mexico | 0.69 | -0.03 | 0.03 | 1.39 | -0.31 | 3.01 | 0.01 | 0.81 | 0.89 | Convex |
| Malaysia | 1.17 | 0.08 | 0.10 | 0.29 | 0.05 | 0.30 | 0.01 | 0.19 | 0.18 | Positive Linear |
| Vanuatu | 0.90 | 0.04 | 0.06 | 0.41 | 0.24 | 0.29 | 0.01 | 0.45 | 0.45 | Positive Linear |
| Nauru | 0.87 | 0.05 | 0.08 | 0.39 | 0.14 | 0.41 | 0.01 | 0.40 | 0.41 | Positive Linear |
| Suriname | 0.50 | -0.03 | 0.01 | 1.75 | 0.26 | 5.41 | 0.01 | 0.82 | 0.87 | Convex |
| Nicaragua | 0.43 | -0.02 | 0.03 | 1.16 | -1.42 | 23.19 | 0.01 | 0.31 | 0.41 | Convex |
| Paraguay | 0.55 | -0.03 | 0.03 | 1.53 | -0.19 | 6.82 | 0.01 | 0.74 | 0.85 | Convex |
| Peru | 0.56 | -0.02 | 0.02 | 1.63 | 0.20 | 5.55 | 0.01 | 0.80 | 0.87 | Convex |
| Papua New Guinea | 0.87 | 0.04 | 0.06 | 0.32 | 0.07 | 0.43 | 0.01 | 0.42 | 0.46 | Positive Linear |
| Pacific Islands (Palau) | 1.02 | 0.05 | 0.08 | 0.32 | 0.04 | 0.35 | 0.01 | 0.60 | 0.67 | Convex |
| Guinea-Bissau | 0.63 | 0.01 | 0.01 | 0.11 | 0.20 | -0.31 | 0.02 | 0.22 | 0.22 | Positive Linear |
| Marshall Islands | 0.95 | 0.06 | 0.09 | 0.37 | 0.17 | 0.22 | 0.01 | 0.54 | 0.55 | Positive Linear |
| Philippines | 1.07 | 0.05 | 0.07 | 0.33 | 0.05 | 0.34 | 0.01 | 0.59 | 0.65 | Convex |
| St. Kitts and Nevis | 0.34 | 0.00 | -0.02 | 0.61 | 1.49 | -9.23 | 0.01 | 0.26 | 0.30 | Positive Linear |
| Senegal | 0.63 | 0.01 | 0.01 | 0.11 | 0.21 | -0.32 | 0.02 | 0.22 | 0.21 | Positive Linear |
| Sierra Leone | 0.58 | 0.01 | 0.01 | 0.18 | -0.05 | 0.87 | 0.01 | 0.47 | 0.55 | Convex |
| St. Lucia | 0.35 | 0.00 | -0.03 | 0.56 | 1.96 | -13.27 | 0.01 | 0.27 | 0.37 | Concave |
| Trinidad and Tobago | 0.46 | -0.04 | 0.01 | 1.69 | -0.06 | 6.55 | 0.01 | 0.84 | 0.94 | Convex |
| Thailand | 1.09 | 0.07 | 0.08 | 0.28 | 0.07 | 0.26 | 0.01 | 0.14 | 0.13 | Positive Linear |
| Tonga | 0.90 | 0.04 | 0.06 | 0.41 | 0.24 | 0.29 | 0.01 | 0.45 | 0.45 | Positive Linear |
| Tunisia | 0.94 | 0.01 | 0.00 | 0.08 | 0.16 | -0.11 | 0.02 | 0.08 | 0.06 | Positive Linear |
| Tuvalu | 0.87 | 0.05 | 0.08 | 0.39 | 0.14 | 0.41 | 0.01 | 0.40 | 0.41 | Positive Linear |
| Uruguay | 0.65 | 0.02 | -0.05 | 0.47 | 2.25 | -4.19 | 0.01 | 0.18 | 0.46 | Concave |
| St. Vincent and the Grenadines | 0.35 | 0.00 | -0.03 | 0.56 | 1.96 | -13.27 | 0.01 | 0.27 | 0.37 | Concave |
| Venezuela | 0.37 | 0.00 | -0.02 | 0.52 | 1.96 | -13.61 | 0.01 | 0.19 | 0.28 | Positive Linear |
| Vietnam | 1.15 | 0.07 | 0.08 | 0.33 | 0.15 | 0.21 | 0.01 | 0.22 | 0.20 | Positive Linear |
| Western Samoa | 0.90 | 0.04 | 0.06 | 0.41 | 0.24 | 0.29 | 0.01 | 0.45 | 0.45 | Positive Linear |
| Malta | 0.97 | 0.01 | 0.00 | 0.11 | 0.14 | -0.03 | 0.02 | 0.18 | 0.15 | Positive Linear |
| New Zealand | 0.81 | 0.04 | 0.03 | 0.37 | 0.50 | -0.33 | 0.01 | 0.26 | 0.24 | Positive Linear |
| Portugal | 1.05 | 0.01 | -0.01 | 0.12 | 0.30 | -0.25 | 0.02 | 0.15 | 0.15 | Positive Linear |
| San Marino | 0.99 | 0.00 | -0.01 | 0.13 | 0.16 | -0.04 | 0.01 | 0.26 | 0.24 | Positive Linear |
| Singapore | 1.13 | 0.08 | 0.10 | 0.25 | 0.00 | 0.31 | 0.01 | 0.12 | 0.11 | Positive Linear |
| Switzerland | 1.05 | 0.01 | -0.01 | 0.11 | 0.33 | -0.30 | 0.02 | 0.10 | 0.13 | Positive Linear |
| United States | 0.80 | -0.01 | 0.06 | 0.64 | -1.32 | 3.73 | 0.01 | 0.34 | 0.49 | Convex |
| New Caledonia | 0.82 | 0.05 | 0.05 | 0.34 | 0.40 | -0.15 | 0.01 | 0.25 | 0.22 | Positive Linear |
| Puerto Rico | 0.34 | 0.00 | -0.02 | 0.61 | 1.49 | -9.23 | 0.01 | 0.26 | 0.30 | Positive Linear |
| Colombia | 0.82 | 0.05 | 0.05 | 0.34 | 0.40 | -0.15 | 0.01 | 0.25 | 0.22 | Positive Linear |
| Central African Republic | 1.15 | 0.07 | 0.09 | 0.32 | 0.16 | 0.19 | 0.01 | 0.21 | 0.19 | Positive Linear |
| Cape Verde | 0.75 | 0.00 | 0.01 | 0.22 | 0.02 | 0.45 | 0.01 | 0.55 | 0.58 | Positive Linear |
| **Expenditure on R&D (% of GDP) and High-technology exports (% of manufactured exports)** | | | | | | | | | | |
| Antigua and Barbuda | 0.54 | 0.08 | 0.09 | 1.41 | 1.19 | 2.27 | 0.01 | 0.29 | 0.26 | Positive Linear |
| Afghanistan | 1.06 | 0.11 | 0.14 | 0.23 | -0.28 | 0.52 | 0.01 | 0.26 | 0.31 | Positive Linear |
| Azerbaijan | 1.14 | 0.10 | 0.13 | 0.25 | -0.23 | 0.47 | 0.01 | 0.29 | 0.33 | Positive Linear |
| Armenia | 1.16 | 0.10 | 0.13 | 0.25 | -0.20 | 0.44 | 0.01 | 0.29 | 0.33 | Positive Linear |
| Argentina | 0.73 | 0.11 | 0.13 | 0.47 | 0.13 | 0.81 | 0.01 | 0.42 | 0.42 | Positive Linear |
| Belize | 0.55 | 0.08 | 0.09 | 1.42 | 0.50 | 8.24 | 0.01 | 0.34 | 0.33 | Positive Linear |
| Bolivia | 0.60 | 0.13 | 0.13 | 0.26 | 0.12 | 0.52 | 0.01 | 0.06 | 0.03 | Positive Linear |
| Solomon Islands | 1.09 | 0.40 | 0.44 | -0.25 | -0.55 | 0.51 | 0.01 | 0.00 | -0.02 | Undefined Complex |
| Brazil | 0.56 | 0.13 | 0.14 | 0.19 | -0.35 | 2.07 | 0.01 | 0.04 | 0.08 | Undefined Complex |
| Chad | 1.02 | 0.11 | 0.08 | 0.27 | 0.65 | -0.36 | 0.01 | 0.33 | 0.32 | Positive Linear |
| Chile | 0.78 | 0.11 | 0.11 | 0.51 | 0.33 | 0.42 | 0.01 | 0.43 | 0.41 | Positive Linear |
| Colombia | 0.55 | 0.09 | 0.08 | 0.93 | 1.89 | -9.28 | 0.02 | 0.13 | 0.11 | Positive Linear |
| Costa Rica | 0.56 | 0.07 | 0.09 | 1.42 | 0.32 | 9.86 | 0.01 | 0.33 | 0.33 | Positive Linear |
| Djibouti | 0.95 | 0.06 | 0.09 | 0.28 | -0.27 | 0.55 | 0.01 | 0.60 | 0.71 | Convex |
| Dominican Republic | 0.52 | 0.08 | 0.09 | 1.35 | 0.78 | 5.78 | 0.02 | 0.24 | 0.22 | Positive Linear |
| Egypt | 1.11 | 0.11 | 0.13 | 0.25 | 0.03 | 0.21 | 0.01 | 0.32 | 0.30 | Positive Linear |
| Eritrea | 1.04 | 0.08 | 0.11 | 0.27 | -0.18 | 0.45 | 0.01 | 0.47 | 0.52 | Positive Linear |
| El Salvador | 0.55 | 0.07 | 0.09 | 1.44 | 0.45 | 8.91 | 0.01 | 0.34 | 0.33 | Positive Linear |
| Ethiopia | 0.87 | 0.05 | 0.09 | 0.29 | -0.44 | 0.72 | 0.01 | 0.70 | 0.85 | Convex |
| Fiji | 1.10 | 0.37 | 0.39 | -0.17 | -0.38 | 0.35 | 0.01 | -0.02 | -0.05 | Undefined Complex |
| Georgia | 1.14 | 0.11 | 0.12 | 0.25 | 0.08 | 0.17 | 0.01 | 0.32 | 0.30 | Positive Linear |
| Guatemala | 0.55 | 0.08 | 0.09 | 1.42 | 0.50 | 8.24 | 0.01 | 0.34 | 0.33 | Positive Linear |
| Honduras | 0.55 | 0.08 | 0.09 | 1.39 | 0.44 | 8.54 | 0.01 | 0.33 | 0.32 | Positive Linear |
| Iran | 1.01 | 0.09 | 0.13 | 0.25 | -0.37 | 0.62 | 0.01 | 0.30 | 0.38 | Positive Linear |
| Iraq | 1.05 | 0.08 | 0.11 | 0.27 | -0.08 | 0.34 | 0.01 | 0.48 | 0.51 | Positive Linear |
| Jamaica | 0.53 | 0.08 | 0.09 | 1.43 | 1.07 | 3.71 | 0.02 | 0.25 | 0.23 | Positive Linear |
| Jordan | 1.08 | 0.09 | 0.12 | 0.26 | -0.09 | 0.34 | 0.01 | 0.43 | 0.46 | Positive Linear |
| Kiribati | 1.03 | 0.27 | 0.21 | 0.13 | 0.62 | -0.76 | 0.01 | -0.01 | 0.03 | Undefined Complex |
| Lebanon | 1.14 | 0.11 | 0.11 | 0.26 | 0.20 | 0.06 | 0.01 | 0.32 | 0.29 | Positive Linear |
| Federated States of Micronesia | 1.16 | 0.37 | 0.46 | -0.03 | -0.65 | 0.69 | 0.01 | -0.03 | 0.03 | Undefined Complex |
| Australia | 1.26 | 0.35 | 0.43 | 0.23 | -0.92 | 2.73 | 0.01 | -0.02 | 0.00 | Undefined Complex |
| Bahrain | 0.97 | 0.07 | 0.10 | 0.27 | -0.24 | 0.51 | 0.01 | 0.50 | 0.58 | Convex |
| Canada | 1.07 | 0.12 | 0.06 | 0.36 | 1.57 | -2.01 | 0.01 | 0.39 | 0.70 | Concave |
| Cuba | 0.53 | 0.08 | 0.09 | 1.43 | 1.07 | 3.71 | 0.02 | 0.25 | 0.23 | Positive Linear |
| Cyprus | 1.13 | 0.12 | 0.13 | 0.25 | 0.15 | 0.09 | 0.01 | 0.31 | 0.29 | Positive Linear |
| Ireland | 1.18 | 0.19 | 0.06 | 0.14 | 0.90 | -0.90 | 0.01 | 0.01 | 0.06 | Undefined Complex |
| Finland | 1.27 | 0.16 | -0.02 | 0.19 | 1.36 | -1.47 | 0.02 | 0.08 | 0.29 | Concave |
| France | 1.19 | 0.15 | 0.07 | 0.18 | 0.71 | -0.68 | 0.01 | 0.08 | 0.13 | Positive Linear |
| Greece | 1.28 | 0.15 | 0.18 | 0.18 | -0.06 | 0.24 | 0.01 | 0.22 | 0.23 | Positive Linear |
| Gaza Strip | 1.13 | 0.11 | 0.12 | 0.25 | 0.06 | 0.19 | 0.01 | 0.32 | 0.30 | Positive Linear |
| Iceland | 1.25 | 0.23 | 0.08 | 0.09 | 1.07 | -1.23 | 0.02 | -0.01 | 0.16 | Concave |
| Israel | 1.13 | 0.11 | 0.12 | 0.25 | 0.06 | 0.19 | 0.01 | 0.32 | 0.30 | Positive Linear |
| Italy | 1.25 | 0.15 | 0.12 | 0.18 | 0.44 | -0.37 | 0.02 | 0.09 | 0.08 | Positive Linear |
| Kuwait | 1.00 | 0.07 | 0.11 | 0.27 | -0.21 | 0.48 | 0.01 | 0.48 | 0.55 | Positive Linear |
| Latvia | 1.21 | 0.14 | 0.03 | 0.16 | 0.93 | -0.97 | 0.02 | 0.14 | 0.31 | Concave |
| Liechtenstein | 1.23 | 0.16 | 0.11 | 0.15 | 0.55 | -0.54 | 0.01 | 0.06 | 0.08 | Positive Linear |
| Monaco | 1.24 | 0.16 | 0.11 | 0.16 | 0.52 | -0.50 | 0.02 | 0.06 | 0.07 | Positive Linear |
| Aruba | 0.55 | 0.10 | 0.08 | 0.91 | 1.82 | -8.82 | 0.02 | 0.13 | 0.11 | Positive Linear |
| American Samoa | 1.10 | 0.37 | 0.39 | -0.17 | -0.38 | 0.35 | 0.01 | -0.02 | -0.05 | Undefined Complex |
| Cayman Islands | 0.55 | 0.08 | 0.09 | 1.42 | 0.50 | 8.24 | 0.01 | 0.34 | 0.33 | Positive Linear |
| Northern Mariana Islands | 1.31 | 0.39 | 0.40 | 0.05 | -0.02 | 0.08 | 0.01 | -0.03 | -0.07 | Undefined Complex |
| French Polynesia | 1.01 | 0.18 | 0.14 | 0.41 | 0.92 | -1.08 | 0.01 | 0.18 | 0.22 | Positive Linear |
| Gibraltar | 1.27 | 0.14 | 0.09 | 0.21 | 0.61 | -0.57 | 0.01 | 0.09 | 0.10 | Positive Linear |
| Guam | 1.34 | 0.39 | 0.38 | 0.12 | 0.17 | -0.06 | 0.01 | -0.02 | -0.06 | Undefined Complex |
| Anguilla | 0.54 | 0.08 | 0.09 | 1.41 | 1.19 | 2.27 | 0.02 | 0.29 | 0.26 | Positive Linear |
| Cook Islands | 0.96 | 0.21 | 0.19 | 0.40 | 0.71 | -0.82 | 0.01 | 0.09 | 0.07 | Positive Linear |
| Jarvis Island | 1.05 | 0.30 | 0.25 | 0.06 | 0.40 | -0.52 | 0.01 | -0.03 | -0.03 | Undefined Complex |
| Falkland Islands (Islas Malvinas) | 0.84 | 0.10 | 0.12 | 0.45 | -0.07 | 1.25 | 0.02 | 0.30 | 0.33 | Positive Linear |
| Baker Island | 1.15 | 0.38 | 0.54 | -0.08 | -1.11 | 1.11 | 0.01 | -0.03 | 0.18 | Convex |
| Guernsey | 1.20 | 0.16 | 0.07 | 0.17 | 0.71 | -0.68 | 0.02 | 0.07 | 0.12 | Positive Linear |
| Guadeloupe | 0.54 | 0.08 | 0.09 | 1.41 | 1.19 | 2.27 | 0.02 | 0.29 | 0.26 | Positive Linear |
| Heard Island & McDonald Islands | 1.06 | 0.02 | 0.07 | 1.06 | 0.19 | 1.83 | 0.01 | 0.48 | 0.48 | Positive Linear |
| Howland Island | 1.15 | 0.38 | 0.54 | -0.08 | -1.11 | 1.11 | 0.01 | -0.03 | 0.18 | Convex |
| Man, Isle of | 1.20 | 0.19 | 0.06 | 0.15 | 0.91 | -0.91 | 0.01 | 0.02 | 0.08 | Undefined Complex |
| Jersey | 1.20 | 0.16 | 0.07 | 0.17 | 0.71 | -0.68 | 0.02 | 0.07 | 0.12 | Positive Linear |
| Jan Mayen | 1.25 | 0.24 | -0.01 | 0.07 | 1.44 | -1.60 | 0.02 | -0.03 | 0.17 | Concave |
| Johnston Atoll | 1.08 | 0.31 | 0.40 | 0.06 | -0.50 | 0.60 | 0.01 | -0.02 | 0.09 | Undefined Complex |
| Montserrat | 0.54 | 0.08 | 0.09 | 1.41 | 1.19 | 2.27 | 0.02 | 0.29 | 0.26 | Positive Linear |
| Midway Islands | 1.14 | 0.39 | 0.54 | -0.10 | -1.11 | 1.10 | 0.01 | -0.02 | 0.18 | Convex |
| Maldives | 1.08 | 0.08 | 0.15 | 1.04 | -0.44 | 3.61 | 0.02 | 0.28 | 0.37 | Positive Linear |
| Mexico | 0.72 | 0.11 | 0.08 | 0.40 | 1.14 | -1.31 | 0.01 | 0.40 | 0.49 | Concave |
| Vanuatu | 1.10 | 0.37 | 0.39 | -0.17 | -0.38 | 0.35 | 0.01 | -0.02 | -0.05 | Undefined Complex |
| Nauru | 1.08 | 0.41 | 0.48 | -0.30 | -0.83 | 0.85 | 0.01 | 0.02 | 0.02 | Undefined Complex |
| Nicaragua | 0.55 | 0.08 | 0.09 | 1.39 | 0.44 | 8.54 | 0.01 | 0.33 | 0.32 | Positive Linear |
| Peru | 0.66 | 0.11 | 0.09 | 0.44 | 1.00 | -2.18 | 0.02 | 0.13 | 0.13 | Positive Linear |
| Panama | 0.52 | 0.09 | 0.08 | 0.90 | 1.27 | -3.53 | 0.02 | 0.14 | 0.11 | Positive Linear |
| Papua New Guinea | 1.24 | 0.36 | 0.35 | 0.10 | 0.19 | -0.15 | 0.01 | -0.03 | -0.07 | Undefined Complex |
| Marshall Islands | 1.11 | 0.40 | 0.60 | -0.13 | -1.39 | 1.33 | 0.01 | -0.01 | 0.29 | Convex |
| Russia | 1.25 | 0.16 | 0.17 | 0.26 | 0.15 | 0.13 | 0.01 | 0.18 | 0.15 | Positive Linear |
| Sudan | 1.00 | 0.09 | 0.10 | 0.27 | -0.03 | 0.30 | 0.01 | 0.54 | 0.55 | Positive Linear |
| Syria | 1.11 | 0.09 | 0.10 | 0.27 | 0.06 | 0.21 | 0.01 | 0.46 | 0.46 | Positive Linear |
| Tonga | 1.10 | 0.37 | 0.39 | -0.17 | -0.38 | 0.35 | 0.01 | -0.02 | -0.05 | Undefined Complex |
| Turkey | 1.15 | 0.11 | 0.12 | 0.25 | 0.14 | 0.11 | 0.01 | 0.33 | 0.31 | Positive Linear |
| Tuvalu | 1.08 | 0.41 | 0.48 | -0.30 | -0.83 | 0.85 | 0.01 | 0.02 | 0.02 | Undefined Complex |
| Turkmenistan | 1.07 | 0.10 | 0.14 | 0.24 | -0.33 | 0.57 | 0.01 | 0.26 | 0.32 | Positive Linear |
| Uruguay | 0.69 | 0.12 | 0.14 | 0.45 | 0.01 | 1.02 | 0.01 | 0.44 | 0.47 | Positive Linear |
| Uzbekistan | 1.07 | 0.10 | 0.14 | 0.24 | -0.33 | 0.57 | 0.01 | 0.26 | 0.32 | Positive Linear |
| Western Samoa | 1.10 | 0.37 | 0.39 | -0.17 | -0.38 | 0.35 | 0.01 | -0.02 | -0.05 | Undefined Complex |
| Yemen | 1.00 | 0.08 | 0.11 | 0.27 | -0.25 | 0.51 | 0.01 | 0.49 | 0.56 | Convex |
| Oman | 0.95 | 0.07 | 0.10 | 0.27 | -0.25 | 0.53 | 0.01 | 0.56 | 0.65 | Convex |
| New Zealand | 0.94 | 0.22 | 0.21 | 0.36 | 0.52 | -0.42 | 0.01 | 0.05 | 0.01 | Undefined Complex |
| Portugal | 1.28 | 0.15 | 0.09 | 0.20 | 0.62 | -0.59 | 0.02 | 0.08 | 0.09 | Positive Linear |
| Qatar | 0.97 | 0.07 | 0.10 | 0.27 | -0.24 | 0.51 | 0.01 | 0.50 | 0.58 | Convex |
| Saudi Arabia | 0.98 | 0.07 | 0.10 | 0.27 | -0.23 | 0.50 | 0.01 | 0.50 | 0.57 | Convex |
| Spain | 1.25 | 0.16 | 0.12 | 0.17 | 0.48 | -0.45 | 0.02 | 0.05 | 0.05 | Positive Linear |
| Switzerland | 1.23 | 0.16 | 0.11 | 0.15 | 0.55 | -0.54 | 0.01 | 0.06 | 0.08 | Positive Linear |
| United Arab Emirates | 0.97 | 0.07 | 0.10 | 0.27 | -0.24 | 0.51 | 0.01 | 0.50 | 0.58 | Convex |
| United Kingdom | 1.20 | 0.19 | 0.06 | 0.15 | 0.91 | -0.91 | 0.01 | 0.02 | 0.08 | Undefined Complex |
| United States | 0.84 | 0.10 | 0.07 | 0.59 | 1.60 | -1.92 | 0.01 | 0.65 | 0.74 | Concave |
| West Bank | 1.12 | 0.11 | 0.12 | 0.26 | 0.08 | 0.17 | 0.01 | 0.32 | 0.30 | Positive Linear |
| New Caledonia | 1.04 | 0.39 | 0.43 | -0.24 | -0.81 | 1.53 | 0.01 | -0.01 | -0.04 | Undefined Complex |
| Puerto Rico | 0.54 | 0.08 | 0.09 | 1.41 | 1.19 | 2.27 | 0.01 | 0.29 | 0.26 | Positive Linear |
| Colombia | 1.04 | 0.39 | 0.43 | -0.24 | -0.81 | 1.53 | 0.01 | -0.01 | -0.04 | Undefined Complex |
| Cape Verde | 1.04 | 0.08 | 0.09 | 0.45 | 0.10 | 0.82 | 0.01 | 0.27 | 0.26 | Positive Linear |
