# Supplementary material for: Global outlook of the multiplier effect of research and development on environmental sustainability
Source: PLoS One. 2023 Sep 21;18(9):e0291370. doi: 10.1371/journal.pone.0291370 (PMC10513248; doi:10.1371/journal.pone.0291370)
Supplement: S1 File — (ZIP) [file pone.0291370.s001.zip › SI Figs 1-7 Permission Letter.docx]

Environmental and Geospatial Sciences Lab

Department of Geography Education

Faculty of Social Sciences

University of Education

P. O. Box 25

Winneba-Ghana

19^th^ May 2023

The Editor

PLOS ONE

**PERMMISION TO USE FIGURES**

I write to give consent for the use of the Figures 1-7 for publication in the paper “Global outlook of the multiplier effect of research and development on environmental sustainability”. The Figures were generated at the Environmental and Geospatial Sciences Laboratory, Department of Geography, University of Education, Winneba-Ghana. The Laboratory gives full permission to publish the Figures under the CC BY 4.0 license.

Thank You

Yours Faithful

Signed

Adams Osman

aosman@uew.edu.gh
